# Understanding the diversity and dynamics of in vivo efferocytosis: Insights from the fly embryo

**DOI:** 10.1111/imr.13266

**Published:** 2023-08-17

**Authors:** Rosalind Heron, Clelia Amato, Will Wood, Andrew J. Davidson

**Affiliations:** ^1^ Institute for Regeneration and Repair University of Edinburgh Edinburgh UK; ^2^ School of Cancer Sciences Wolfson Wohl Cancer Research Centre, University of Glasgow Glasgow UK

**Keywords:** chemotaxis, *Drosophila*, in vivo imaging, inflammation, monocytes/macrophages, phagocytosis

## Abstract

The clearance of dead and dying cells, termed efferocytosis, is a rapid and efficient process and one that is critical for organismal health. The extraordinary speed and efficiency with which dead cells are detected and engulfed by immune cells within tissues presents a challenge to researchers who wish to unravel this fascinating process, since these fleeting moments of uptake are almost impossible to catch in vivo. In recent years, the fruit fly (*Drosophila melanogaster)* embryo has emerged as a powerful model to circumvent this problem. With its abundance of dying cells, specialist phagocytes and relative ease of live imaging, the humble fly embryo provides a unique opportunity to catch and study the moment of cell engulfment in real‐time within a living animal. In this review, we explore the recent advances that have come from studies in the fly, and how live imaging and genetics have revealed a previously unappreciated level of diversity in the efferocytic program. A variety of efferocytic strategies across the phagocytic cell population ensure efficient and rapid clearance of corpses wherever death is encountered within the varied and complex setting of a multicellular living organism.

## 1 INTRODUCTION

Throughout our lifetime, the body removes unwanted and potentially harmful material through programmed cell death. This is most abundant during embryogenesis, when the embryo initially produces an abundance of cells and then selectively eliminates them to sculpt the different organs. Dead cells must be removed by the remaining healthy cells of the tissue, which internalize the cellular debris through engulfment. Programmed cell death and the associated cell clearance continues to be important after birth in order to maintain tissue homeostasis and health, since the process of engulfment is also required for the clearance of dead cells arising in an unpredictable manner through injury and disease. While many cells have a basal ability to clear cellular debris, many multicellular organisms have evolved specialized cells dedicated to this role, known as phagocytes. While phagocytes can engulf a variety of particles, the clearance of dead and dying cells is specifically known as “efferocytosis”. Efferocytosis as a process is highly conserved throughout evolution, from fruit flies to humans, which allows researchers to utilize more simplistic models to investigate the underlying mechanisms of this fascinating and fundamental aspect of biology.

In this review, we will highlight the contribution of the fruit fly embryo in advancing our understanding of efferocytosis in vivo. In particular, we will demonstrate that efferocytosis is not the homogeneous process that it is often considered to be. In fact, we now know that this is an incredibly diverse process, complicated by an ever‐increasing number of distinct ways for cells to die, disparate phagocytes, distinct modes of engulfment, redundant efferocytic receptors, and divergent pathways for corpse degradation. The genetic tractability of *Drosophila* makes it substantially easier and faster to identify and study underlying molecular pathways than it would be in mammals. For this reason, the fly has been used as a model for biological research for over a century. In fact, to date, six Nobel Prizes in Physiology or Medicine have been awarded to 10 researchers that have made their ground‐breaking discoveries using *Drosophila*. Seminal work on the *Drosophila* embryo in particular revealed the Nobel prize winning role of the segmentation genes in defining the dorsal‐ventral body axis.^1^ Furthermore, one of the developmental genes identified as part of these embryonic screens, *toll*, ultimately led to another Nobel prize for its distinct role in pathogen recognition in immunity.^2^ As such, the humble fruit fly remains a genetic powerhouse in current biomedical research.

The diversity and complexity of efferocytosis makes it difficult to fully recapitulate in vitro, necessitating in vivo models. Furthermore, precise examination of this highly dynamic process demands live imaging. As such, these conflicting requirements make the study of efferocytosis a challenging undertaking. *Drosophila* is the simplest genetic model that possesses professional phagocytes. By utilizing the translucent fly embryo, the phagocytes can be visualized in vivo and in real‐time, capturing their role in the clearance of the pronounced cell turnover associated with development. Furthermore, the embryo can also be readily and reproducibly wounded, allowing for the study of how phagocytes clear dead cells within damaged tissue. Combined with the unrivaled genetics of the fly, the *Drosophila* embryo is perfectly positioned to unveil the role of phagocytes in clearing dead and dying cells. Here, we bring together a wealth of research that has used *Drosophila* embryos to contribute to our recent understanding of the process of efferocytosis, following the cell corpse in a journey from its death to its degradation within the innards of a phagocyte. At each stage of this journey, we will highlight the diversity inherent to efferocytosis in all its complex glory.

### 1.1 Diversity in cell death

**FIGURE 1:**
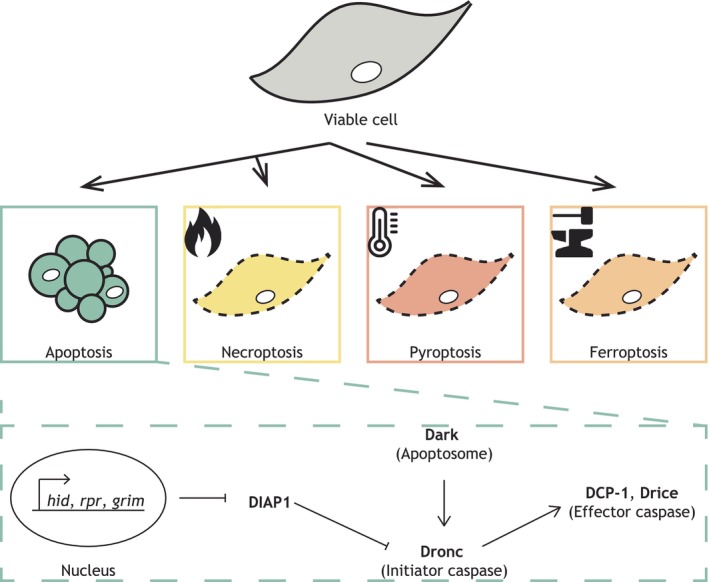
Diversity of cells death modalities. A viable cell (gray) can die by different means, including apoptosis (green) and multiple types of necrosis, such as necroptosis (yellow), pyroptosis (dark orange) and ferroptosis (light orange). An overview of the apoptotic signaling pathway is shown (dashed green rectangle). At the onset of apoptosis, Dark (main component of the apoptosome) activates the initiator caspase Dronc, which in turn leads to the activation of the effector caspase DCP‐1. In the presence of apoptotic triggers, expression of *hid*, *rpr* and *grim* leads to degradation of DIAP1, which in turn allows Dronc activation.

Cell death is synonymous with life and is required for normal development and homeostasis as well as occurring during infection and disease. Indeed, abnormally high levels of cell death are the ultimate causative agent of pathology. Regardless of whether cell death occurs in sickness or health, it is phagocytes that are enlisted to clear the resulting cellular debris. Originally, it was believed that cells could only die by either programmed apoptosis or accidental necrosis.^3^ However, recently, many more distinct cell death modalities have been identified.^4^ While many of these different forms of cell death have yet to be directly visualized in vivo, it is already clear that there are far more ways for a cell to die than originally appreciated (Figure 1). This diversity undoubtedly presents a huge challenge to the phagocytes tasked with clearing the dead cells, especially since many modes of cell death arise in an unpredictable way (e.g., in response to unexpected injury). Therefore, phagocytes must be highly versatile in order to maintain their crucial clearance function in the face of so many different types of cell death.

The *Drosophila* embryo has long served as a powerful model to study cell death and how it impacts the remaining healthy tissue left behind. Ultimately, the *Drosophila* embryo provides a genetically tractable and optically translucent in vivo setting in which to interrogate different modes of cell death and their clearance by phagocytes. In this section, we will highlight the diverse range of different types of cell death and how *Drosophila* and, in particular, the *Drosophila* embryo have contributed to our understanding of them.

#### 1.1.1 Apoptotic cell death

Apoptosis is a programmed form of cell death, wherein the cell undergoes a deliberate and highly orchestrated demise.^4^ It can be induced by a wide range of different cellular stresses, including tissue damage, infection, and oncogenic transformation, but also as a normal part of organismal development and tissue homeostasis. As such, apoptosis has a wide range of molecular triggers. However, all these apoptotic pathways converge on the caspases, proteolytic enzymes which evolved with the specific purpose of promoting a programmed cell death.^5^ Caspase activation instigates the cleavage of a multitude of intracellular targets, which lead to a controlled demolition of the cell. Ultimately, nuclear condensation and cellular fragmentation break the apoptotic cell down into small, self‐contained apoptotic bodies, a process that is generally assumed to aid clearance by phagocytes.

While these cell autonomous aspects of apoptosis can be readily recapitulated in vitro, the full role of apoptosis is only evident when considered within the context of a living organism. When apoptosis occurs within a tissue, it is actively extruded by its healthy neighbors, again presumably to aid engulfment.^6^ Furthermore, the apoptotic cell can release factors to stimulate regenerative proliferation (termed “apoptosis‐induced proliferation”) in order to ensure replacement of the lost cell and thus maintain tissue integrity.^7^, ^8^, ^9^ Once extruded from the tissue, the apoptotic corpse is cleared via efferocytosis, often through the recruitment of professional phagocytes such as macrophages.^10^ Finally, even after engulfment, the apoptotic corpse can influence the behavior of the phagocyte it is internalized within. For example, the uptake of apoptotic corpses by macrophages is known to alter the behavior of these cells making them less inflammatory and more regenerative (“M2‐like polarization”).^11^, ^12^ Thus, apoptosis can directly stimulate regeneration, through release of proliferative factors, or indirectly, through modulation of the phagocyte they are internalized within. These attributes mean apoptosis has conventionally been considered “inflammatory‐silent”, whereby cell death does not trigger an aggressive recruitment of immune cells as seen during necrotic tissue damage. Although it has recently been shown that some forms of apoptosis can be “immunogenic”, developmental apoptosis is undoubtedly non‐inflammatory, so as to avoid damaging the very tissues the developing organism is endeavoring to sculpt.^4^ Nevertheless, apoptosis is still actively cleared by phagocytes, including immune cells, wherein cells such as macrophages are recruited to engulf the cellular debris.^10^ The importance of this engulfment is underlined by autoimmune pathologies, such as lupus, where accumulating, uncleared apoptotic corpses trigger chronic inflammation. Aging apoptotic cells will eventually lose their cellular integrity and become necrotic, a process known as “secondary necrosis”.^13^ Therefore, the timely clearance of apoptotic debris is a crucial aspect of the ability of apoptosis to kill cells without triggering inflammation.

*Drosophila melanogaster*, alongside *Caenorhabditis elegans*, has contributed enormously to our understanding of in vivo apoptotic cell death. For example, the study of the developmental apoptosis occurring during *Drosophila* embryogenesis revealed the transcriptional control of apoptosis via the Reaper family of genes.^14^ The delineation of how the expression of these genes induced programmed cell death converged with the discovery of proteins that actively inhibit apoptosis (Figure 1). Such proteins were originally identified in viral pathogens, which suppressed apoptosis in order to avoid elimination of infected cells by the host.^15^ One such protein identified from baculovirus was named Inhibitor of Apoptosis (IAP). Subsequently, a *Drosophila* homologue of IAP was discovered, named DIAP1, and loss of *diap1* resulted in catastrophic apoptosis within the whole *Drosophila* embryo.^16^, ^17^ It quickly became evident that the viral IAP had evolved to mimic the role of DIAP during infection to prevent infection‐induced apoptosis, aiding viral replication and dissemination.^16^ DIAP acts as a failsafe mechanism to prevent unintentional activation of apoptosis, as this constitutive inhibition must be relieved to allow apoptosis to proceed.^18^, ^19^ DIAP1 achieves its anti‐apoptotic role by targeting the initiator caspase Dronc for degradation, ensuring that activated Dronc cannot accidently initiate the caspase cascade.^18^, ^20^ This inhibition is overcome through Reaper family expression, which target DIAP1 for degradation, allowing sufficient Dronc for activation. This failsafe anti‐apoptotic mechanism is conserved in mammals, where IAP proteins have also been identified.^21^ Although regulation of mammalian IAPs has shifted from the nucleus to the mitochondria, the mechanism is otherwise highly conserved.^22^, ^23^ As such, *Drosophila* Reaper expression is sufficient to trigger apoptosis in mammalian cell culture.^24^, ^25^

Apoptosis can be classified as extrinsic or intrinsic, based on whether it is receptor‐ or mitochondrially activated.^4^ While extrinsic apoptosis has not been as extensively studied in the fly, there are strong parallels between fly and mammals in caspase activation during intrinsic apoptosis (Figure 1). In both, it is the apoptosome that acts as a platform to activate the initiator caspases, which triggers the caspase cascade. In *Drosophila*, the APAF‐1 homologue, Dark, forms the main structure of the apoptosome.^26^ However, a key difference between fly and mammalian intrinsic apoptosis is the lack of a requirement for mitochondrial release of cytochrome c for apoptosome assembly in the former, which is consistent with the nuclear control of *Drosophila* apoptosis.^27^, ^28^ However, curiously, *Drosophila* possess two well‐conserved BCL‐2 homologs.^29^, ^30^, ^31^ In mammals, BCL‐2 family proteins can be both pro‐ and anti‐apoptotic, and regulate the release of pro‐apoptotic factors (including cytochrome c) from the mitochondria in order to initiate intrinsic apoptosis.^5^ *Drosophila* embryos mutant for both BCL‐2 family homologs exhibit no abnormality in developmental apoptosis, which is consistent with a lack of mitochondrial control of apoptosis in the fly.^32^ Therefore, quite why *Drosophila* possess a BCL‐2 family remains a mystery. Confusingly, mammalian BCL‐2 family members appear functional in *Drosophila*, whereby pro‐apoptotic mammalian Bax induces apoptosis when expressed in the fly embryo.^33^ It is possible *Drosophila* BCL‐2 family members nonessentially support apoptosis, contribute to certain types of stress‐induced apoptosis, or are involved in unrelated mitochondrial remodeling.^34^ Alternatively, mitochondrial permeabilization contributes to many other distinct modes of cell death.^4^ Due to the overriding role of the BCL‐2 family in mammalian apoptosis, it has been difficult to untangle the role of these proteins in non‐apoptotic forms of regulated cell death. Therefore, *Drosophila* may prove a powerful model to cleanly dissect the role of BCL‐2 family members in different modes of cell death. Furthermore, as our appreciation of programmed necrosis and its different inflammatory outputs has grown, there is a real need for genetically tractable models to explore these diverse death modalities in vivo. With its unrivaled genetics, excellent optical properties for in vivo imaging and ease with which necrosis can be triggered through laser‐ablation, the *Drosophila* embryo could serve as a powerful platform to meet this need.

#### 1.1.2 Necrotic cell death

Historically, necrosis was considered an unregulated event, wherein cell lysis caused cellular swelling and the uncontrolled release of intracellular contents. Many of the factors released during necrosis are known to trigger inflammation and drive the recruitment of immune cells, even in the absence of pathogens. As such, and in stark contrast to apoptosis, necrosis had long been considered an accidental and undesirable cell death, little more than a physical rupturing of the cell.^3^ However, more recently, it has been discovered that necrosis can be triggered in a regulated way. In fact, many, distinct forms of regulated necrosis have been identified, each driven by different (although sometimes overlapping) toxic cellular processes.^4^ In some cases, the extent of regulation even reaches what could be defined as “programmed”, wherein molecular pathways have evolved with the specific purpose of inducing a defined mode of necrosis.^5^ Examples of these include necroptosis and pyroptosis, which both diverge off from the caspase cascade. Other forms of regulated necrosis do not quite reach this definition (or at least not yet) and are somewhere in‐between.^5^ An example is ferroptosis, caused by the overwhelming accumulation of lipid peroxidation.^35^, ^36^ While pathways exist to detoxify lipid peroxidation and therefore prevent ferroptosis, an initiator with the specific purpose of triggering ferroptosis has not yet been clearly identified.^37^, ^38^, ^39^ More and more molecularly defined ways to kill cells in vitro are being discovered and proposed as new forms of regulated cell death.^4^ What has lagged behind is direct proof of their existence in vivo, especially live cell imaging demonstrations. This is primarily due to a lack of probes and genetic tools to specifically induce, label and manipulate a given form of necrosis.

The *Drosophila* embryo has served as a powerful model to generate necrotic tissue damage. Laser ablation can be used to generate sterile necrotic wounds in order to study both the inflammatory and wound healing responses.^40^ Importantly, these wounds are entirely necrotic, exhibiting no evidence of the caspase activity that characterizes apoptosis.^41^ As will be discussed in later sections, this necrosis is highly inflammatory, resulting in the immediate influx of macrophages, which are recruited to the wound to clear the necrotic debris.^40^ However, until recently, it has only ever been possible to visualize this necrotic tissue damage indirectly, delineated by the closing wound edge and the encircling macrophages. Lacking a means to directly label the necrosis, the wound itself has remained a “black void” despite its central role in orchestrating this sterile inflammation. Recently, we and others have been able to microinject fluorescent necrotic dyes into the embryo in order to reveal the necrosis within these wounds.^42^, ^43^ Furthermore, the use of the far‐red necrotic dye, DRAQ7, has allowed us to visualize this necrosis through three‐color, live imaging.^43^ This dye rapidly enters lysed cells and is a general necrotic marker rather than distinguishing any specific form of regulated necrosis. While it is likely that the labeled necrosis at the center of the wound is unregulated necrosis, caused by laser‐induced lysis of the cells, it remains entirely possible that there are other, distinct forms of necrosis occurring during such acute tissue damage. For example, mathematical modeling of calcium signaling in the immediate aftermath of wounding in the *Drosophila* pupa has predicted the requirement for both immediate and slower necrosis.^44^, ^45^ Necrotic tissue damage results in protease release, the enzymatic activity of which ultimately triggers the latter of two calcium waves through the surrounding tissue, which is needed for inflammation. A delayed form of necrosis, with a longer, slower release of proteases, was determined to be necessary to sustain this calcium signaling. Moreover, calcium signaling is known to act upstream of reactive oxygen species (ROS) generation in wounded *Drosophila* embryos.^46^ The high levels of ROS present in the wounds of both *Drosophila* and vertebrates could contribute to other forms of regulated cell death.^47^, ^48^ For example, the combination of ROS and iron can fuel the Fenton chemistry that drives lipid peroxidation, the causative agent of ferroptosis.^4^ It has been demonstrated that there is a ferroptotic component to mammalian tissue damage, including murine models of kidney ischemia/reperfusion injury.^38^, ^49^ While lipid peroxidation has been implicated in inflammatory signaling emanating from zebrafish wounds, and the presence of ferroptosis has been hinted at in various *Drosophila* models, there has been no direct in vivo visualization of ferroptosis in wounded *Drosophila* embryos or any other model.^50^, ^51^, ^52^

The existence of programmed necrosis in *Drosophila* is even less clear. *Drosophila* lacks identifiable homologs of gasdermins, which are the pore‐forming proteins acting downstream of inflammatory caspases during pyroptosis. However, it has been recently shown that even bacteria have a functional gasdermin ortholog, which is sufficient to induce bacterial cell death.^53^ *Drosophila* also have several understudied caspases, with the possibility that these are the equivalent of the inflammatory caspases.^54^, ^55^ However, this remains in the realm of speculation and awaits further study. As for necroptosis, one study has suggested the existence of a *Drosophila* necroptotic pathway, which branches off the initiator caspase Dronc.^56^ Interestingly, the role of Dronc in triggering this cell death was independent of its caspase activity and the resulting cell death was necrotic, as visualized by the uptake of necrotic dyes. However, *Drosophila* lacks an ortholog of MLKL, the activity of which induces necroptosis.^4^ Interestingly, the conservation of MLKL, even across very closely related mammals, is highly variable.^57^ Therefore, it remains possible that there are other implementers of necroptosis. As such, *Drosophila* may possess a rudimentary form of necroptosis or, alternatively, this may represent some other form of programmed necrosis.

#### 1.1.3 Autophagic and lysosomal cell death

Autophagic cell death is a distinct form of cell death that is associated with autophagy.^4^ While there is currently no evidence of it occurring in the embryo, autophagic cell death is well characterized in the *Drosophila* salivary glands.^58^ The giant cells that make up this larval gland die at the onset of metamorphosis, exhibiting numerous autophagosomes. Inhibition of either autophagy or caspases suppresses cell death in the metamorphosing salivary gland, whereas combined inhibition of both strongly promotes survival.^58^ This implies an autophagic and an apoptotic component acting in unison during salivary gland cell death. It is interesting to speculate on the prominent role of autophagic cell death in metamorphosis, whereby autophagy may be deployed to recoup as many nutrients as possible from the turning over tissue. However, as there has not yet been a role identified for autophagic cell death in *Drosophila* embryogenesis, we will limit our discussion of this mode of cell death. However, its existence further testifies to the diversity of cell death faced by phagocytes during development.

Similarly, lysosomal cell death is not currently known to occur in the *Drosophila* embryo, but has a prominent role in developmental cell death within the *Drosophila* egg chambers.^59^ The large nurse cells of the egg chamber provide nutrients to the developing oocyte, climaxing with cytoplasmic dumping of the contents of the nurse cells into the growing oocyte. Afterward, the remains of the nurse cells are removed through cell death and clearance by follicle cells, the semi‐professional phagocytes acting in the “immune‐privileged” egg chamber.^60^ While caspases and autophagy contribute to this developmental cell death, the combined inhibition of both did not completely block nurse cell removal.^61^ Interestingly, developmental nurse cell death required acidification, implicating lysosomal cell death.^59^ Again, we will limit our discussion of lysosomal cell death, so as to focus on the embryo. However, of particular note, many distinct types of cell death occur within the *Drosophila* egg chambers, both during development and in response to stresses such as starvation (reviewed in detail here^62^). As such, this tissue alone highlights the variety of cell death, which ultimately presents different challenges to the phagocytes tasked with clearing the remains.

### 1.2 Diversity of phagocytes

**FIGURE 2:**
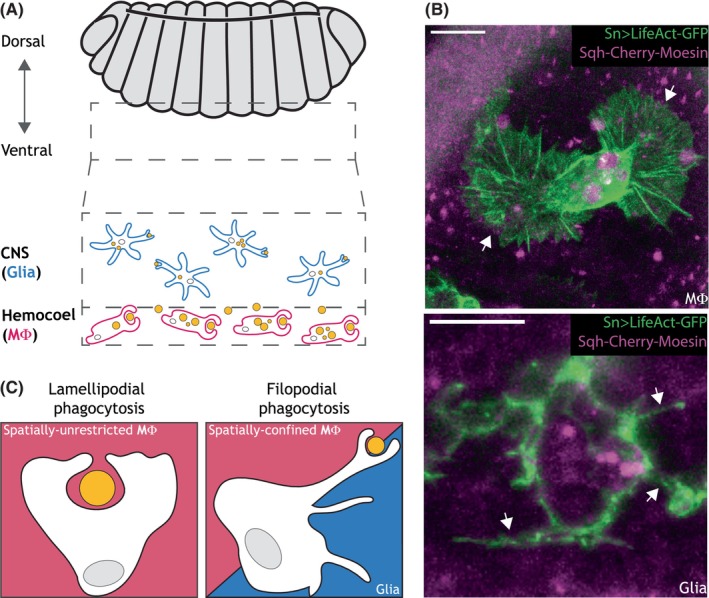
Diversity of phagocytic cells and modes of engulfment. (A) Schematic representation of a stage 15 *Drosophila* embryo (gray). The ventral aspect is highlighted by the dashed gray rectangle, and the CNS‐resident glia (cyan) and macrophages (magenta) indicated. Apoptotic corpses are shown in yellow. (B) Representative images from a super resolution, in vivo, real‐time video showing an embryonic macrophage (top) and a glial cell (bottom) expressing an F‐actin marker (green) and an apoptotic corpse marker (magenta). Arrowheads indicate the lamellipod extended by the macrophage and the filopods extended by the glia, respectively. Scale bars represent 10 mm. (C) Schematic representation of lamellipodial phagocytosis (left, magenta box) by a spatially‐unrestricted macrophage, and filopodial phagocytosis (right) typically extended by a spatially‐confined macrophage (magenta half) or a CNS‐resident glia (cyan half).

The high volume of developmental apoptosis occurring within the *Drosophila* embryo is cleared by two distinct phagocytes: the macrophage‐like embryonic plasmatocytes and the phagocytic glia of the central nervous system (Figure 2).^63^ In this section, we will describe these different cell types, compare and contrast their efferocytic behavior, and highlight how the study of the *Drosophila* embryo has contributed to our appreciation of the diversity of in vivo cell death and its clearance.

#### 1.2.1 The distinct phagocytes of the *Drosophila* embryo

The embryonic plasmatocytes represent the vast majority of the hemocytes, which fulfill the same role as the white blood cells in higher eukaryotes. The plasmatocytes are the functional equivalents of the macrophage and will be referred to as such for the rest of this review. Like their mammalian counterparts, *Drosophila* macrophages play a vital role in clearing the cellular debris generated during development, both during embryogenesis and metamorphosis. These two distinct developmental stages sculpt (and then re‐sculpt) the organism into two contrasting entities. Unsurprisingly, both life stages are associated with a high burden of developmental apoptosis, the clearance of which is a critical function of the macrophages. These important cells also serve as the immune sentinels of the fly and, as such, rapidly respond to tissue damage in order to clear necrotic debris and pathogenic invaders at sites of insult. Consequently, these macrophages are extremely phagocytic. Furthermore, in contrast to larval and adult stages, where the macrophages are passively pumped around the body in the blood‐like “hemolymph”, both embryonic and pupal macrophages exhibit high motility.^64^, ^65^, ^66^

In the embryo, macrophages originate from the head mesoderm and migrate along three main routes during embryonic development to populate the animal.^67^, ^68^, ^69^ The macrophages that traverse around the dorsal side of the embryo have an arduous migration, requiring invasion into the germ band.^70^, ^71^, ^72^ While this dorsal migration likely involves the clearance of apoptotic corpses, the majority of studies on embryonic efferocytosis has been conducted on the ventral side of the embryo due to the ease of imaging and high levels of apoptosis (Figure 2). Here, the macrophages migrate along the length of the developing central nervous system (CNS), before spreading out to populate the entire ventral side of the embryo.^68^, ^73^ This ventral migration is exquisitely choreographed to coincide with a wave of apoptosis during mid‐late embryogenesis.^67^, ^74^, ^75^ The majority of embryonic apoptosis occurs in the developing CNS, with up to 30% of neurons dying through apoptosis.^76^, ^77^, ^78^ As the macrophages disperse through an interstitial cavity between the outermost epithelium and the CNS, they actively engulf this developmental debris. The dispersal of the macrophages is largely independent of this apoptosis and instead is orchestrated by a series of PDGF/VEGF (Pvf) ligands expressed along the midline.^68^ However, it has been demonstrated that the absence of apoptosis can lead to subtle changes to macrophage dispersal.^42^

These ventrally placed macrophages cannot penetrate deeper into the CNS and are limited to clearing the apoptosis at the interface between their interstitial cavity and the developing nerve cord.^78^ Instead, as in mammals, *Drosophila* has tissue‐resident phagocytes that clear debris within the “immune‐privileged” CNS. In the *Drosophila* embryo, these are the phagocytic glia (Figure 2). Glia is an umbrella term for all of the non‐neuronal cells in the nervous system that play supporting roles for the neurons, including the clearance of unwanted neuronal debris. In vertebrates, phagocytosis in the CNS is primarily performed by specialist, tissue‐resident macrophages known as the microglia (non‐glial, despite the name), with help from glial cells called astrocytes.^79^, ^80^, ^81^, ^82^, ^83^ *Drosophila* does not have microglia, so the phagocytic functions in the CNS are performed solely by the tissue‐resident phagocytic glia.^78^, ^84^, ^85^, ^86^ Although not macrophage in origin, vertebrate microglia and *Drosophila* phagocytic glia are similar molecularly, morphologically and physiologically.^87^, ^88^, ^89^, ^90^ These cells are generally classified as “semi‐professional phagocytes”; however, this term greatly undersells their phagocytic capability. Like the macrophages, phagocytic glia are more active during the developmental stages of embryogenesis and metamorphosis, when more cell clearance is required. However, unlike the macrophages, phagocytic glia are immotile and remain resident in the nervous system throughout all stages of life. Accumulating evidence shows that the phagocytic activity of these glia is comparable to that of macrophages, and consequently, these cells deserve to be considered as a second professional phagocyte in *Drosophila*.^78^, ^91^, ^92^, ^93^, ^94^, ^95^

The macrophages and phagocytic glia of the *Drosophila* embryo co‐operate to clear apoptosis within the developing CNS (Figure 2). This is underscored by the overburdening of macrophages in embryos that lack all glia.^96^ However, the macrophages and the embryonic glia are strikingly different phagocytes. Most obviously, the macrophages are highly motile cells, which actively seek out and migrate toward apoptotic corpses, even over long distances.^43^ In contrast, the phagocytic glia are immotile and embedded within the embryonic CNS.^78^

**FIGURE 3:**
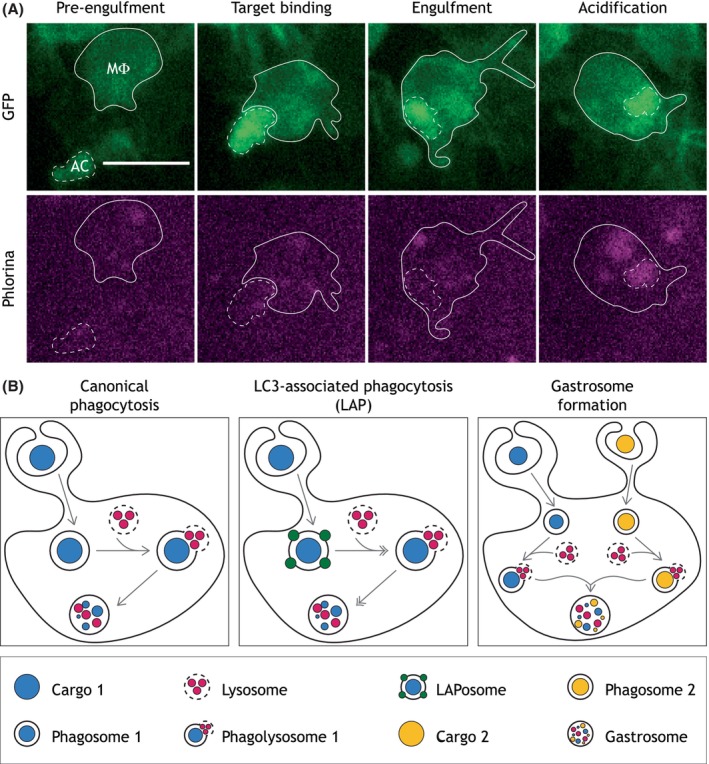
Real‐time tracking of apoptotic corpses and diversity of corpse processing. (A) Representative images from in vivo, real‐time video of a *Drosophila* embryonic macrophage (MF) and a developmentally‐induced, CharON labeled apoptotic corpse (AC) prior to their interaction (Pre‐engulfment), during the initial contact (Target binding), upon internalization (Engulfment) and processing (Acidification). GFP (top panel) labels both the MF and the AC as it undergoes apoptosis and is engulfed. Phlorina (magenta, bottom panel) labels the AC once it has been acidified within the phagolysosome of the phagocyte. Scale bar represents 10 mm. (B) During canonical phagocytosis (left), the internalized cargo fuses to a lysosome to form a phagolysosome, where degradation occurs. During LC3‐Associated Phagocytosis (LAP, center), LC3 decorates the phagosome, setting it on a fast‐track for lysosomal fusion and degradation. During gastrosome formation (right), phagolysosomes originating from distinct events of engulfment converge within a single compartment.

A crucial advance in our ability to track efferocytosis has been recently made thanks to the generation of transgenic flies expressing a novel biosensor, named CharON.^43^ This probe combines a caspase‐activated GFP, which specifically labels apoptosis, and a pH‐sensitive red fluorophore, which exhibits increased fluorescence within the acid environment of the phagolysosomes. Development of this novel, genetically encoded biosensor provided the first opportunity to visualize all stages of efferocytosis in vivo, from apoptosis, through recognition and uptake of the corpse, to its final digestion (Figure 3). Through the use of CharON, the global pattern of efferocytosis within the fly embryo has now been analyzed. Efferocytosis deep within the CNS is stochastic throughout mid‐late embryogenesis, indicative of a steady rate of continuous uptake by the phagocytic glia. In contrast, the clearance at the hemocoel‐CNS interface rapidly increases at the same developmental stage, reflecting a defined wave of efferocytosis by the dispersing macrophages.

The differential identity of phagocytic glia is distinguished from the macrophages by the expression of the homeodomain transcription factor *repo*.^97^ However, these distinct phagocytes express a similar suite of efferocytic receptors. For example, macrophages and phagocytic glia express the Nimrod‐family receptors SIMU and Draper, which are utilized by both phagocytes during the clearance of developmental apoptosis.^42^, ^78^, ^98^ The role of these phagocytic receptors in corpse uptake and processing will be discussed in more detail in Section 1.3. However, of important note here is the differential regulation of these receptors within these phagocytes during the acquisition of their phagocytic identity. For example, the expression of SIMU and Draper in phagocytic glia is controlled by the key regulator of glial cell fate Repo.^94^ Furthermore, FGF receptor signaling is another recently identified pre‐requisite for establishing the efferocytic identity of these phagocytic glia.^99^ In contrast, the upregulation of Draper in embryonic macrophages is triggered by exposure of the naïve macrophages to apoptosis as they first emerge during their ventral dispersal. More specifically, it is the uptake of the initial apoptotic corpse that increases Draper expression and triggers maturation, or “priming”, of the embryonic macrophages.^41^ Furthermore, engulfment also appears to drive upregulation of the efferocytic receptor Croquemort.^100^ Corpse uptake by macrophages triggers a transient increase in intracellular calcium within the engulfing cell, which leads to increased c‐Jun N‐terminal kinase (JNK) signaling and culminates in the increased expression of Draper.^41^ While the expression of SIMU and Draper is hard wired into phagocytic glia and does not require priming, their upregulation also appears to involve JNK signaling, in this case, acting downstream of Repo.^101^ It is interesting to consider why embryonic macrophages require priming, whereas the phagocytic glia do not. One potential explanation lies in the different patterns of efferocytosis revealed by CharON discussed above.^43^ The phagocytic glia exhibit a constant rate of stochastic efferocytosis throughout mid‐late embryogenesis. In contrast, macrophage efferocytosis occurs within an extremely acute developmental window, triggered when they emerge on the ventral side of the embryo and are first confronted with a dense field of apoptosis. Therefore, it is possible that the initial exposure to apoptosis is a useful cue for the need to rapidly upregulate their suite of efferocytic receptors.

Glia also utilize their phagocytic machinery for the clearance of axonal and dendritic branches as well as excessive presynaptic inputs during neuronal and synaptic pruning.^102^, ^103^, ^104^ These processes are essential for the formation and strengthening of functional neuronal circuits in the developing CNS. Evidence from glial phagocytosis during metamorphosis in the pupa shows that the efferocytic pathways utilized by glia are dependent on the nature of the neuronal debris to be cleared. More specifically, distinct phagocytic pathways are activated for the removal of pruned neurites compared to the clearance of apoptotic neuronal cell bodies, even if they both derive from the same neuron. For instance, Draper is essential for clearance of neuronal cell bodies, while elimination of neurites solely requires Crk/Mbc/Ced12 signaling.^85^

Despite their obvious differences, the phagocytes of the embryo share many similarities, and comparing and contrasting their efferocytic behavior has greatly advanced our understanding of the clearance of developmental apoptosis. Furthermore, the unique in vivo context provided by the *Drosophila* embryo has offered the opportunity to explore how diverse phagocytes co‐operate to fulfill their critical clearance function.

#### 1.2.2 Clearance of necrosis within the embryo

Phagocytic cells are also important for the clearance of necrotic debris following tissue damage. When a wound is created in the epithelium of the *Drosophila* embryo, macrophages rapidly migrate toward the injury site and phagocytose the resulting cellular debris.^41^, ^105^ While wounds can still close in the absence of macrophages, these phagocytes are required for the clearance of the necrotic debris and any invading pathogens.^40^ Interestingly, if the embryo is wounded before the developmental dispersal of macrophages is complete, the macrophages will ignore the wound and prioritize the clearance of developmental apoptosis.^48^ This is due to the fact that, as mentioned above, embryonic macrophages cannot recognize and respond to inflammatory stimuli (e.g., wounds) until they have engulfed apoptotic debris,^41^ meaning that developmentally‐induced apoptosis is required for sensitizing macrophages to necrotic tissue damage. Of important note, too much apoptosis is as detrimental to inflammation as too little, as the high levels of uncleared apoptosis in *simu* mutant embryos distract macrophages from responding to wounds.^42^ Interestingly, there is no difference in wound recruitment between wildtype macrophages carrying a high load of internalized apoptotic corpses versus those carrying a low burden.^43^ This might suggest that the chemotactic factors released by the uncleared apoptotic corpses (“Find Me” signals) within the *simu* mutant compete with the inflammatory factors released at wounds.^43^ Alternatively, the aging, uncleared apoptosis may well be undergoing secondary necrosis and confounding the inflammatory recruitment of macrophages to acute injury.

Although the exact chemoattractant that guides macrophages to wounds is still to be determined, hydrogen peroxide release during wounding is important for triggering the inflammatory response, from *Drosophila* to humans.^41^, ^46^, ^47^, ^106^, ^107^ In the fly, this is detected by a redox‐sensitive cysteine within the src family kinase Src42a, which then phosphorylates Draper.^84^ Draper activation is associated with receptor clustering, which involves the recently identified inflammatory regulator Pez.^108^, ^109^ This activation of Draper leads to the recruitment of the downstream effector kinase Shark.^98^ While the precise details remain uncertain, Shark activity then feeds into the cytoskeletal machinery to promote guided migration of the macrophage toward the wound.

The phagocytic glia have not yet been implicated in the clearance of necrosis during embryonic wounding. However, in the adult fly, glia utilize Draper to engulf debris in response to damaged neurons as well as during neuronal pruning.^84^, ^102^ Interestingly, an inhibitory splice variant of Draper (Draper‐II) is also expressed in phagocytic glia after the recruitment of these cells to severed axons and acts in a feedback loop to suppress Draper activity.^110^ This is understood to prevent excessive and damaging glial activity in the CNS. This same feedback loop has also been implicated in macrophages of the *Drosophila* embryo, whereby increased expression of Draper‐II in the macrophages impaired inflammatory migration to wounds.^98^

#### 1.2.3 Phagocyte heterogeneity

Recently, *Drosophila* immune cells have been extensively profiled by single‐cell RNA sequencing, revealing interesting heterogeneity across the cell population.^111^, ^112^, ^113^, ^114^ However, these studies were restricted to larval stages of development and, to date, the analysis of embryonic immune cells has been limited to bulk RNA sequencing, highlighting their developmental role as opposed to their anti‐microbial specialization required in the larva.^111^ Another study used an alternative approach to identify different macrophage subsets in the embryo,^115^ but whether this represents truly distinct cell types or rather plasticity amongst the one population of embryonic macrophages, remains unclear. In a separate study, through the use of the efferocytic biosensor CharON, it was recently demonstrated that embryonic macrophages exhibit a striking heterogeneity in the final number of apoptotic corpses internalized per cell.^43^ While this might suggest different subpopulations of macrophages, with differing phagocytic ability, computational modeling suggested otherwise. In fact, it appears that all embryonic macrophages possess a near equal efferocytic potential, an extremely high phagocytic capacity, and essentially compete for the available apoptotic corpses. Increased apoptotic corpse burden did not impair the recruitment of wildtype macrophages to necrotic wounds, but, interestingly, a high corpse burden did suppress further uptake of necrotic debris once at sites of tissue damage. The same study showed longer‐term adaption of macrophages to high corpse burden, consistent with macrophages being highly plastic rather than sub‐specialized.

Less is known regarding heterogeneity amongst the phagocytic glia of the embryo. Furthermore, our knowledge is hampered by a poor understanding regarding the developmental connection between the phagocytic glia of the larvae and the efferocytic glia in later life stages. For example, the secondary astrocyte‐like and ensheathing glial cells that form during the late larval stages are also phagocytic.^116^, ^117^ These cells are important for efferocytosis during metamorphosis and, in fact, they become the main phagocytic cells within the CNS of the pupa and adult. These differ from phagocytic cells in the *Drosophila* embryo in that they do not express SIMU. Draper, however, remains crucial for the process of efferocytosis at this stage.^95^, ^110^, ^118^ Whether the origins of these secondary astrocyte‐like glia can be identified, and what becomes of the phagocytic glia of the embryo at later stages, remains unclear. However, given the diversity of the glia and their vital and exclusive role in supporting the neurons, it is highly likely that other subtypes of glia are also efferocytic.

#### 1.2.4 Other *Drosophila* phagocytes

While the *Drosophila* embryo currently has only two established phagocytes, later life stages of the fly possess some truly diverse phagocytes, which will be briefly highlighted here. While later life stages possess distinct, very large hemocytes (known as lamellocytes) for encapsulating parasitic wasp eggs, these have not been shown to engulf cellular debris. As introduced in Section 1.1, the *Drosophila* ovary provides a unique insight into the interaction between phagocytes and dying cells. Like the CNS, the *Drosophila* ovary is considered “immune‐privileged”, with macrophages largely excluded. In this environment, it is the phagocytic follicle cells that clear dying nurse cells, relying on the same efferocytic pathways as the glia and the macrophages, including Draper and dCED12.^60^, ^119^, ^120^ However, as discussed in Section 1.1, nurse cells use distinct methods, including a death that is not entirely dependent on apoptosis nor autophagy.^61^ Instead, the follicle cells seem to use their phagocytic machinery to actively induce the death of nurse cells in a process referred to as “phagoptosis”.^119^ The exact method by which the follicle cells trigger death of nurse cells is not yet clear, but it appears to require the lysosomal pathway.^61^, ^119^ Although phagoptosis has not been demonstrated in other phagocytic cells within the fly, these studies using follicle cells demonstrate that phagocytes can play a far more active role than previously thought in inducing the cell death that they are tasked with clearing.

Another distinct and unexpected phagocyte within the fly is the fat body cell, the *Drosophila* equivalent of the liver and adipose tissue combined. Surprisingly, live imaging in the *Drosophila* pupa revealed that fat body cells become motile and can actively migrate toward wounds.^121^ Furthermore, these cells migrate using a strikingly different method to macrophages, utilizing an adhesion‐independent, actomyosin‐driven, peristaltic mode of movement. Interestingly, once recruited to the wound, the fat body cells co‐operate with macrophages to clear necrotic cell debris. Whether these cells are capable of clearing developmental apoptosis remains an open question.

### 1.3 Diversity of engulfment

Different phagocytes have a predisposition to clear cellular debris in certain morphologically distinct ways, determined by their specific characteristics. For example, the phagocytic glia of the *Drosophila* embryo are immobile and so cannot utilize protrusive‐based motility to move toward and envelop apoptotic corpses they are charged with clearing.^78^ Furthermore, the microenvironment the phagocytes reside in also dictates the phagocytic behavior that is achievable. For instance, the aforementioned embryonic glia are embedded within the developing CNS, where space to move and engulf debris is severely limited.^78^ Phagocytes have evolved to function within the distinct tissues they inhabit, requiring diverse phagocytic behaviors and strategies. However, this tissue‐specific context is impossible to fully replicate in vitro and is also difficult to capture using any other technique than live imaging. As such, the *Drosophila* embryo offers a unique model with which to dissect in vivo phagocytosis.

#### 1.3.1 Phagocytes exhibit distinct modes of engulfment

As introduced in Section 1.2 and discussed above, the phagocytic glia of the *Drosophila* embryo clear apoptosis from the developing CNS.^78^ They are embedded within a tightly packed tissue, where they are, unsurprisingly, immobile. Morphologically, they are highly spiky cells, closely resembling the microglia, which are the resident macrophages of the vertebrate CNS.^122^, ^123^ Furthermore, like the microglia, the *Drosophila* phagocytic glia appear to use their spiky, “filopodial” protrusions to reach out and grasp cellular debris (Figure 2).^78^

While embryonic glial phagocytosis appears to be morphologically homogeneous, engulfment throughout embryogenesis can be far more diverse, as exhibited by the very dynamic and multi‐functional macrophages. On the ventral side of the *Drosophila* embryo, these professional phagocytes clear developmental apoptosis arising at the interface between the hemocoel (the interstitial “blood‐cavity”) and the underlying CNS.^43^, ^67^ Like many motile cells, macrophages move by extending large, sheet‐like protrusions known as lamellipods (Figure 2).^40^ These highly dynamic structures are extended through actin polymerization, which is mediated by the actin nucleator Arp2/3 complex.^124^, ^125^ The Arp2/3 complex is recruited and activated at the leading edge of cells by the nucleation‐promoting factor SCAR (also known as “WAVE”).^126^ The retrograde flow of this actin away from the cell edge is what propels the cell forward.^127^ The dendritic network of polymerized actin generated by the Arp2/3 complex is then remodeled into linear, cross‐linked actin bundles, which act to reenforce and enhance lamellipod extension.^128^ A distinct actin nucleator, Ena (also known as “VASP”) acts upstream of actin cross‐linkers such as Fascin and Fimbrin during bundling of the actin network within the lamellipod.^129^, ^130^ Ena acts at the protrusive edge of the cell, capturing the growing ends of branching actin networks and elongating them processively, allowing the actin bundlers to cross‐link the filamentous actin into bundles.^130^, ^131^ These same actin bundles also underlie the extension of thin, spiky protrusions called filopods, which can be generated by Ena or by another actin nucleator, Dia.^132^, ^133^ Together, the Arp2/3 complex, Ena and Dia can be considered the principal actin nucleators within *Drosophila*, whereby the former generates the branched actin underlying lamellipod formation, while the other two create linear actin bundles found within lamellipods and filopods.

The embryonic macrophages appear to primarily utilize their Arp2/3 complex‐dependent lamellipods to move toward and envelop apoptotic debris, presumably due to their inherent motility (Figure 2). However, it had been noted that these macrophages could also engulf apoptotic corpses through the use of protrusions that are more filopodial in morphology.^41^ This was of particular interest due to work in mammalian macrophages demonstrating Arp2/3 complex‐independent phagocytosis, up ending decades of conventional thinking.^134^ The molecular mechanism of engulfment in the absence of the Arp2/3 complex remained uncertain. However, the low cytoskeletal redundancy of *Drosophila* combined with its powerful genetics, offered a unique opportunity to investigate distinct forms of macrophage phagocytosis. Furthermore, the in vivo setting, wherein macrophages can be studied within the context of a developing embryo, allowed an exploration of why macrophages possess different modes of engulfment.

As observed in mammalian macrophages, *Drosophila* macrophages with suppressed Arp2/3 complex activity were still capable of particle uptake.^105^, ^134^, ^135^ These macrophages lack lamellipods and exhibit poor motility, adopting a spiky morphology resembling that seen in many different models.^136^, ^137^, ^138^ Nevertheless, these macrophages could maintain their critical clearance function by reaching out with phagocytic filopods and drawing material back into their immobile cell body.^105^ These phagocytic filopods were also occasionally observed during wildtype macrophage engulfment, particularly during the early stages of their developmental dispersal. These filopods were projected toward their target in a highly directed manner, implying they could detect and be guided toward the cellular debris. We concluded that macrophages possess two morphologically distinct modes of engulfment, which we named “lamellipodial phagocytosis” and “filopodial phagocytosis” (Figure 2). Consistent with their established roles in filopod formation, both Ena and Dia localized to the phagocytic filopods of Arp2/3 complex‐deficient macrophages. Furthermore, the combined inactivation of both Ena and Dia was sufficient to suppress phagocytosis in Arp2/3 complex‐deficient macrophages. This implied that lamellipodial phagocytosis and filopodial phagocytosis were not merely morphologically distinct, but were also distinct at the molecular level. However, incredibly, the combined loss of all three of the principle nucleators within *Drosophila* was not sufficient to fully abolish all phagocytosis. This remarkable robustness suggests there may yet be other modes of engulfment available to macrophages, and is a testament to the cytoskeletal plasticity of these phagocytes.

Having established lamellipodial phagocytosis and filopodial phagocytosis as distinct modes of engulfment, the immediate question became why did macrophages require different means of taking up cellular debris. The independence of filopodial phagocytosis from motility was one telling difference, allowing immobile phagocytes to reach cellular debris that were not in their immediate proximity. Consistent with this hypothesis, the non‐motile phagocytic glia, which are buried within the embryonic CNS, utilize filopodial phagocytosis.^43^, ^78^ However, the embryonic macrophages are highly motile, so why might they resort to filopodial phagocytosis? The breakthrough came through the use of the developmental *slit* mutant, wherein the cavity through which the macrophages disperse fails to open correctly.^139^ In this mutant, the macrophages that had forced themselves the furthest around the blocked embryo were engulfing debris through filopodial phagocytosis.^105^ This suggested spatial constraint could be one reason that macrophages might switch to utilizing filopodial phagocytosis. Indeed, when fluorescent dextran was injected into wildtype embryos to visualize the extracellular space surrounding macrophages during their developmental engulfment, macrophages adopted filopodial phagocytosis when they were spatially constrained. More specifically, when a macrophage encountered an apoptotic corpse located at the bottom of a narrow, dextran‐filled channel, it would extend a phagocytic filopod to grab this otherwise unreachable debris. Therefore, we concluded that macrophages resorted to filopodial phagocytosis to overcome the spatial constraint frequently encountered within their complex environment in vivo. Again, this was consistent with the filopodial phagocytosis exhibited by the phagocytic glia embedded within the compact CNS.^78^ Another established reason for cells adopting a filopod‐dominated, dendritic morphology includes tissue surveillance, which allows sentinel resident macrophages to monitor a large area without resorting to motility.^122^, ^140^ This behavior can allow tissue‐resident macrophages within the mouse to respond to limited “micro‐lesions”, without triggering full‐scale inflammation and the collateral damage this entails.^141^ Furthermore, the phagocytosis of particles varying in size and opsonization differed in their requirement for the Arp2/3 complex.^134^ This suggests that distinct forms of engulfment are best‐suited for different target particles.

In the *Drosophila* embryo, the macrophages exhibit a remarkable ability to dynamically switch between lamellipodial and filopodial phagocytosis to fulfill their critical clearance function within the “obstacle course” encountered in vivo.^105^ This underscores the phagocytic plasticity required by macrophages and goes some way to explain the necessity of the cytoskeletal robustness, wherein macrophages can blend the activities of different actin regulators to generate a diverse range of protrusive shapes. Furthermore, this work highlights the strengths of the *Drosophila* embryo for providing an in vivo context to better understand macrophage behavior.

#### 1.3.2 Phagocytes possess a diverse repertoire of efferocytic receptors

Not only do phagocytes exhibit distinct modes of engulfment, but they also possess a complex collection of efferocytic receptors. In mammalian cells, this represents a truly bewildering array of phagocytic receptors with overlapping activities (reviewed in Refs. [142, 143]). This redundancy has rendered the investigation of mammalian efferocytic receptors problematic. These receptors can be broadly divided into three types. “Tethering receptors” lack significant intracellular domains and help the phagocyte engage with the corpse during engulfment. In contrast, “docking receptors” possess substantial intracellular domains, which trigger internal signaling to promote engulfment. “Bridging molecules” are secreted proteins that opsonize cellular debris and are subsequently recognized by the other efferocytic receptors to facilitate phagocyte‐corpse engagement. *Drosophila* also possess numerous phagocytic receptors and their individual and collective functions remain far from clear. However, the reduced redundancy and genetic tractability of the fly offers a foothold to address this challenge. In particular, the *Drosophila* embryo and the clearance of developmental apoptosis therein, has been used to identify receptors required for efferocytosis. Notably, the discovery of the CD36 homologue Croquemort, and its requirement for macrophage‐mediated uptake of apoptosis, was an early demonstration of the power of using the *Drosophila* embryo and its phagocytes to study efferocytosis.^100^ Integrins have also be implicated in *Drosophila* efferocytosis, consistent with their role in mammalian engulfment.^144^ However, it is the Nimrod superfamily that contains the largest collection of *Drosophila* phagocytic receptors.^145^, ^146^ Several members of this family are involved in the macrophage‐mediated clearance of microbial pathogens.^147^ However, long before they were grouped within the Nimrod family, Draper and SIMU had been implicated in the engulfment of cellular debris in multiple *Drosophila* phagocytes.^42^, ^60^, ^78^, ^148^ Both of these receptors have been reported to bind phosphatidylserine, the classic “Eat‐me” signal exposed on the outer membrane of apoptotic corpses.^149^, ^150^ However, these two receptors have very different intracellular domains. Like Croquemort, SIMU, has an extremely limited intracellular domain and is considered a tethering receptor.^78^ In contrast, Draper has a substantial intracellular domain and can be considered a docking receptor. Interestingly, within the *Drosophila* embryo, Draper is not essential for macrophage or glial apoptotic corpse uptake and instead appears necessary for subsequent corpse processing.^78^, ^98^ However, this is not clear cut and Draper does appear to contribute to corpse uptake in other contexts and developmental stages.^148^, ^151^, ^152^ Nevertheless, for the phagocytes of the embryo, it is SIMU that appears to be more generally required for apoptotic corpse uptake.^78^ Given the absence of any intracellular domain in SIMU, there is presumably a need for as yet unidentified docking receptor(s) to promote corpse uptake. Furthermore, SIMU has been implicated in retaining macrophages at necrotic tissue damage in wounded *Drosophila* embryos.^42^ Since necrotic cells do not actively expose phosphatidylserine and there is no apoptosis induced during wounding of the embryo, it is unclear how SIMU contributes to this process.

The Nimrod family member NimB4 has been identified as a secreted bridging molecule necessary for efficient clearance of apoptotic corpses in *Drosophila* embryos and larval brains.^151^ NimB4 was found to bind apoptotic corpses and enhances efferocytosis, but not necessary for phagocyte‐corpse engagement, consistent with it acting as a bridging molecule. The precise efferocytic receptor(s) NimB4 interacts with to aid uptake remains unknown. Furthermore, this work also revealed a role for the microbe‐clearing NimC1 and Eater in apoptotic corpse uptake, further complicating our understanding of efferocytic receptors in *Drosophila*. Finally, the chemokine‐like Orion was recently identified as another bridging molecule, which supports the interaction of Draper with phosphatidylserine during clearance of dying neurons.^153^

As observed in mammals, *Drosophila* phagocytes possess a diverse repertoire of efferocytic receptors with overlapping activities. Collectively, all these receptors undoubtedly help the different phagocytes fine tune their efferocytic ability to help them accommodate the wide range of cell death they can encounter in vivo. Furthermore, these different receptors might drive distinct forms of engulfment to help phagocytes clear the same type of debris in drastically different tissue environments. There is much still to learn on this complicated topic. However, with its many highlighted advantages, the *Drosophila* embryo offers a powerful platform to help tackle this challenge.

### 1.4 Diversity in corpse processing

Our current understanding of how engulfed apoptotic corpses are processed has in part been inferred from studies on pathogen clearance conducted in *Drosophila* as well as in other in vivo and in vitro systems. Simply put, there are two key events that newly‐formed phagosomes must undergo to ensure effective degradation of their content. Firstly, they must fuse to pre‐existing endosomes and lysosomes. Secondly, they must progressively acidify their lumen pH.

Fusion events allow exposure of the phagosome content to a variety of degradative enzymes (e.g., cathepsin and DNAses). As these degradative enzymes are not initially present in the newly‐formed phagosome, fusion enables their introduction to the engulfed material. The different fusion steps (from tethering to early endosomes to formation of phagolysosomes) are mediated by different members of the Rab family, notably Rab5 and Rab7, which sequentially localize to the phagosome membrane.^154^ While the role of the Rabs on pathogen clearance has been assessed in *Drosophila* macrophages,^155^ far less is currently known about their role in efferocytosis, particularly during embryonic development. However, *Drosophila* embryonic macrophages expressing a dominant negative version of Rab5 appear more vacuolated than their wildtype counterparts,^135^ strongly supporting a role for Rab5 in apoptotic corpse degradation in flies.

Another crucial step for cargo degradation is the progressive reduction of the phagolysosome lumen pH. Acidification allows lysosomal hydrolases to reach their peak enzymatic activity while also weakening the interaction between internalized ligands and receptors, so that the latter can be effectively recycled back to the plasma membrane. Acidification is mediated by a large protein complex termed the Vacuolar ATPase (V‐ATPase), which pumps H^+^ across the phagosome membrane into the lumen in an ATP‐dependent fashion. To date, while the role of V‐ATPase during pathogen clearance in *Drosophila* macrophage‐like S2 cells has been explored, its involvement in corpse processing has yet to be shown.^156^, ^157^

As for the ultimate fate of acidified apoptotic corpses, again, little is currently known about the steps that follow phagolysosome formation in fly embryonic macrophages. Pioneering work on the highly phagocytic single celled organism *Dictyostelium*, later supported by observations in cultured mammalian macrophages, suggests that a late neutralization phase takes place.^158^, ^159^ The increase in pH is mediated by the actin nucleation‐promoting factor WASH (encoded by *washout* in the fly), which actively removes the V‐ATPase from the phagolysosome membrane, therefore arresting the proton influx. Potential evidence in support of the occurrence of a neutralization phase in *Drosophila* comes from a study using cultured fly macrophages fed with pHrodo‐labeled bacteria, which exhibit increasing fluorescence intensity in progressively acidic cellular compartments. Unlike controls, WASH‐depleted cells maintain a steadily increasing fluorescence emission profile,^160^ suggesting that, in the absence of WASH, phagolysosomes may not undergo neutralization. Interestingly, despite the complete collapse of the endosomal system in *wash‐*deficient mammalian cell lines, *washout* is not essential for fly viability.^160^, ^161^ Therefore, the precise role of WASH in *Drosophila* remains unresolved.

#### 1.4.1 Efferocytic receptors contribute to corpse degradation

As emphasized in Section 1.3, how *Drosophila* embryonic macrophages process engulfed apoptotic corpses is still far from fully elucidated. There is, however, substantial evidence supporting a role for a number of phagocytic receptors belonging to the Nimrod superfamily during efferocytosis in the developing embryo.

Interestingly, despite being the homologue of the canonical efferocytic receptor, CED‐1, Draper is not required for corpse uptake in either the macrophages or the phagocytic glia of the embryo.^78^, ^98^ Instead, Draper has been implicated in corpse processing due to at least two lines of evidence. Firstly, macrophages expressing a mutated version of Draper or subjected to RNAi‐mediated reduction of endogenous Draper levels appear hypervacuolated, suggesting impaired corpse turnover.^98^ Secondly, macrophages expressing a mutated version of Draper fail to achieve phagolysosome formation, as demonstrated by the lack of co‐localization between LysoTracker and Lamp1 (phagosomal and lysosomal markers respectively).^151^ Importantly, Draper has also been shown to mediate apoptotic debris clearance by *Drosophila* embryonic glia, but does so downstream of the tethering receptor SIMU.^78^ More specifically, loss of *simu*, but not *draper*, impaired apoptotic corpse uptake by phagocytic glia within the embryonic CNS. However, double *simu*; *draper* mutant glia resembled *simu* mutants and lacked internalized corpses, implying SIMU acts upstream of Draper during engulfment. More recently, NimB4, another member of the Nimrod superfamily and a secreted bridging molecule, has been shown to regulate apoptotic corpse processing in *Drosophila* embryonic macrophages and in the larval glia.^151^ NimB4 has been demonstrated to control the fusion step between phagosomes and lysosomes, and its loss remarkably recapitulates the phenotype observed upon loss of Draper function.^151^ To date, why different efferocytic receptors are required for corpse processing rather than corpse recognition remains unclear.

#### 1.4.2 Alternative corpse degradation pathways

Adding to the glaringly fragmented knowledge of how *Drosophila* embryonic phagocytic cells process engulfed material is the steadily increasing evidence that these cells possess many more degradation pathways than originally appreciated. In fact, over the last few years, novel pathogen and cell death processing modes have been uncovered. Here we briefly focus on two of them: LC3‐Associated Phagocytosis (LAP) and gastrosome formation (Figure 3).

LAP entails the conjugation of LC3/Atg8 on single membrane vesicles originated from events of phagocytosis.^162^ Its discovery upturned the long‐standing notion that LC3 is exclusively involved in autophagy and solely found on double‐membrane intracellular vesicles known as autophagosomes. Despite obvious molecular overlaps, LAP differs from autophagy. For instance, the ULK1/Atg1‐containing pre‐initiation complex is only strictly required for the latter to occur,^163^ whilst Rubicon and Atg16L1 are absolutely essential only for LAP to take place.^164^, ^165^ LAP has been demonstrated to occur in mammalian macrophages both in vivo and in vitro, and to modulate their inflammatory behavior during clearance of dead cells/pathogens and tumor progression.^162^, ^163^, ^164^, ^165^, ^166^

The term gastrosome refers to a single and morphologically unique vesicle that has recently been described in zebrafish microglia and shown to accommodate the content of multiple mature phagolysosomes.^167^ Much about the gastrosome has still to be unveiled, yet the potential implications of its existence are great. For instance, given the fact that the gastrosome appears to be enriched in membranes and lipids and that its size and overall morphology have been shown to affect microglia functionality,^167^ gastrosome abnormalities may underpin a wide range of human diseases. So far, LAP and the gastrosome have been observed in experimental systems other than the fly (although excitingly, while this review was in revision, LAP was demonstrated to be required in glia for the removal of axonal debris in clipped *Drosophila* wings).^168^ Understanding whether they occur in *Drosophila* phagocytic cells, during embryogenesis or later developmental stages, would provide an additional and valuable experimental set‐up to further explore their dynamics and functional relevance in vivo.

Ultimately, whilst underscoring the incompleteness of our knowledge, the discovery of LAP and the gastrosome prompts us to ask why phagocytic cells may have evolved different routes to degrade engulfed corpses. To date, it is widely accepted that targeting LC3 to phagosomes sets ingested material on a fast‐track for degradation, highlighting the remarkable ability of phagocytic cells to assign a “priority code” to engulfed particles.^162^ However, crucially, what dictates the urgency in processing ingested material in vivo remains to be determined at this stage. Equally, gastrosome discovery challenges the longstanding dogma that phagolysosomes are a terminal compartment, the ultimate cradle of processed material, once again highlighting how fragmented our understanding of corpse processing still is. While the existence of a gastrosome in *Drosophila* has yet to be investigated, it is worth pointing out that fusion events between pre‐existing phagolysosomes containing an apoptotic corpse and newly‐internalized necrotic debris have recently been reported in macrophages at embryonic wounds.^43^ Whether this is strictly a gastrosome remains to be shown, but this finding demonstrates that fly macrophages also possess an intracellular compartment where the content of multiple phagosomes can converge.

#### 1.4.3 The unresolved outcome of engulfed corpses

While many fundamental aspects of corpse processing in *Drosophila* embryonic phagocytic cells remain far from elucidated, it appears clear that the fly embryo provides a perfect experimental system to dissect the process of efferocytosis in vivo.

A powerful example of how the *Drosophila* embryo can help us interrogate and unravel the complexity and diversity of corpse clearance in vivo is the newly‐engineered CharON probe (Figure 3).^43^ From a corpse processing point of view, CharON has made a series of intriguing observations possible. For instance, we now know that corpse acidification occurs at a significantly slower rate than the initial phagocytosis and that the presence of apoptotic corpses in the process of being degraded does not prevent macrophages from engulfing additional apoptotic debris. This implies that embryonic macrophages are capable of “prioritizing” the removal of developmental apoptosis, swiftly clearing even the most densely packed field of apoptosis, presumably to prevent delayed clearance and the accompanying risk of inflammatory secondary necrosis. Interestingly, macrophages that have engulfed many apoptotic corpses can adapt to their high burden with increased corpse acidification, suggesting an increased rate of degradation. Interestingly, *Drosophila* embryonic macrophages must also upregulate their cytoprotective defenses to protect against the elevated ROS generated during the degradation of these engulfed apoptotic corpses. This highlights that the clearance of this cellular debris presents a significant challenge even long after its uptake.^169^

Many questions arise from these newly‐found answers. Future research should aim to understand how the phagocytic burden within an individual macrophage can influence macrophage identity and/or behavior. In fact, it has long been known that recognition and engulfment of dying cells has a profound impact on the inflammatory behavior of macrophages,^12^, ^170^ but whether the number of ingested apoptotic corpses can modulate macrophage function in vivo has only just begun to be explored.^43^ Another interesting open question is how long macrophages hold on to their corpse burden, and what the ultimate fate of the engulfed material is. To address some of these points, the ability to live image macrophages in vivo beyond embryogenesis, into later developmental stages, becomes paramount. Given the fundamental importance of these questions, the technical challenges to overcome will likely be worth the effort.

## 2 CONCLUSION

There has been a tendency to dismiss efferocytosis as a mundane and uninteresting task, an afterthought to the cell death process. However, it is now clear that in vivo efferocytosis, with its intrinsic diversity, represents a significant challenge to the phagocytes tasked with clearance.

The efferocytic heterogeneity exists at every step of this process. For instance, we now know that cell death itself is extremely varied, with different modalities posing unique challenges to the engulfing cells. Furthermore, the purpose and circumstances of the cell death also vary, including developmental apoptosis, cell turn over during homeostasis and tissue damage during acute injury and pathology. To ensure successful clearance of this diverse cell death in a manner that is appropriate to the tissue context, phagocytes must be highly plastic. For example, the clearance of apoptosis during development must be inflammatory silent to ensure no collateral damage to the nascent tissue. In contrast, the clearance of necrotic debris at wounds, relying heavily on the recruitment of inflammatory cells, often comes at the cost of further damage to the tissue.^141^ While excessive phagocyte activity has its costs, it is also true that failure to clear cell death underpins different pathologies, including chronic inflammation and autoimmunity. Further underscoring the diversity of efferocytosis in vivo is the existence of distinct phagocytes, which have undoubtedly evolved to adapt to the staggering complexity and variety of the tissues that exist in vivo. These phagocytes possess multiple means of engulfing cellular debris, conferring them with additional phagocytic flexibility. In order to fulfill their critical clearance function under all the complex and varied circumstances encountered in vivo, phagocytes possess a highly dynamic cytoskeleton and a wide array of different efferocytic receptors. These mediate an extremely sophisticated interaction between phagocytes and corpses, and often work beyond simple uptake, shepherding the debris through the subsequent degradation process. Lastly, internalized corpses can be degraded in multiple ways involving very different intracellular pathways. Crucially, the uptake of debris subsequently influences the behavior of the phagocyte, with a change in any one of the above highlighted factors capable of completely changing the phagocytes' identity and response. Ultimately, whichever aspect of this process we study, we are confronted with an ever‐increasing diversity and complexity, often leaving us with more questions than answers. Nevertheless, we are undeniably advancing our understanding of cell death and its clearance, greatly aided by simpler in vivo models such as the *Drosophila* embryo. Thanks to its genetic tractability and live imaging potential, the fly continues to uncover unexpected diversity in the efferocytic program in vivo, from mechanisms of clearance to the types of cells that can carry out this critical function. Future live cell imaging studies in the *Drosophila* embryo are likely to continue to implement our understanding of this fascinatingly complex process, finding very much needed answers to the many open questions that remain.

## CONFLICT OF INTEREST STATEMENT

The authors declare no conflicts of interest or financial interests.

## Data Availability

The data that support the findings of this study are available from the corresponding author upon reasonable request.

## References

[1] Nusslein‐Volhard C , Wieschaus E . Mutations affecting segment number and polarity in Drosophila. Nature. 1980;287(5785):795‐801. 6776413 10.1038/287795a0

[2] Lemaitre B , Nicolas E , Michaut L , Reichhart J‐M , Hoffmann JA . The dorsoventral regulatory gene cassette spätzle/toll/cactus controls the potent antifungal response in drosophila adults. Cell. 1996;86(6):973‐983. 8808632 10.1016/s0092-8674(00)80172-5

[3] Kerr JF , Wyllie AH , Currie AR . Apoptosis: a basic biological phenomenon with wide‐ranging implications in tissue kinetics. Br J Cancer. 1972;26(4):239‐257. 4561027 10.1038/bjc.1972.33 PMC2008650

[4] Galluzzi L , Vitale I , Aaronson SA , et al. Molecular mechanisms of cell death: recommendations of the nomenclature committee on cell death 2018. Cell Death Differ. 2018;25(3):486‐541. 29362479 10.1038/s41418-017-0012-4 PMC5864239

[5] Green DR . Cell Death: Apoptosis and Other Means to an End. 2nd ed. Cold Spring Harbor Laboratory Press; 2018.

[6] Rosenblatt J , Raff MC , Cramer LP . An epithelial cell destined for apoptosis signals its neighbors to extrude it by an actin‐ and myosin‐dependent mechanism. Curr Biol. 2001;11(23):1847‐1857. 11728307 10.1016/s0960-9822(01)00587-5

[7] Medina CB , Mehrotra P , Arandjelovic S , et al. Metabolites released from apoptotic cells act as tissue messengers. Nature. 2020;580(7801):130‐135. 32238926 10.1038/s41586-020-2121-3 PMC7217709

[8] Huh JR , Guo M , Hay BA . Compensatory proliferation induced by cell death in the Drosophila wing disc requires activity of the apical cell death caspase Dronc in a nonapoptotic role. Curr Biol. 2004;14(14):1262‐1266. 15268856 10.1016/j.cub.2004.06.015

[9] Ryoo HD , Gorenc T , Steller H . Apoptotic cells can induce compensatory cell proliferation through the JNK and the wingless signaling pathways. Dev Cell. 2004;7(4):491‐501. 15469838 10.1016/j.devcel.2004.08.019

[10] Wood W , Turmaine M , Weber R , et al. Mesenchymal cells engulf and clear apoptotic footplate cells in macrophageless PU.1 null mouse embryos. Development. 2000;127(24):5245‐5252. 11076747 10.1242/dev.127.24.5245

[11] Voll RE , Herrmann M , Roth EA , Stach C , Kalden JR , Girkontaite I . Immunosuppressive effects of apoptotic cells. Nature. 1997;390(6658):350‐351. 9389474 10.1038/37022

[12] Fadok VA , Bratton DL , Konowal A , Freed PW , Westcott JY , Henson PM . Macrophages that have ingested apoptotic cells in vitro inhibit proinflammatory cytokine production through autocrine/paracrine mechanisms involving TGF‐beta, PGE2, and PAF. J Clin Invest. 1998;101(4):890‐898. 9466984 10.1172/JCI1112 PMC508637

[13] Silva MT , do Vale A , dos Santos NM . Secondary necrosis in multicellular animals: an outcome of apoptosis with pathogenic implications. Apoptosis. 2008;13(4):463‐482. 18322800 10.1007/s10495-008-0187-8 PMC7102248

[14] White K , Grether ME , Abrams JM , Young L , Farrell K , Steller H . Genetic control of programmed cell death in Drosophila. Science. 1994;264(5159):677‐683. 8171319 10.1126/science.8171319

[15] Bump NJ , Hackett M , Hugunin M , et al. Inhibition of ICE family proteases by baculovirus antiapoptotic protein p35. Science. 1995;269(5232):1885‐1888. 7569933 10.1126/science.7569933

[16] Hay BA , Wassarman DA , Rubin GM . Drosophila homologs of baculovirus inhibitor of apoptosis proteins function to block cell death. Cell. 1995;83(7):1253‐1262. 8548811 10.1016/0092-8674(95)90150-7

[17] Wang SL , Hawkins CJ , Yoo SJ , Muller HA , Hay BA . The Drosophila caspase inhibitor DIAP1 is essential for cell survival and is negatively regulated by HID. Cell. 1999;98(4):453‐463. 10481910 10.1016/s0092-8674(00)81974-1

[18] Meier P , Silke J , Leevers SJ , Evan GI . The Drosophila caspase DRONC is regulated by DIAP1. EMBO J. 2000;19(4):598‐611. 10675329 10.1093/emboj/19.4.598 PMC305598

[19] Goyal L , McCall K , Agapite J , Hartwieg E , Steller H . Induction of apoptosis by Drosophila reaper, hid and grim through inhibition of IAP function. EMBO J. 2000;19(4):589‐597. 10675328 10.1093/emboj/19.4.589 PMC305597

[20] Wilson R , Goyal L , Ditzel M , et al. The DIAP1 RING finger mediates ubiquitination of Dronc and is indispensable for regulating apoptosis. Nat Cell Biol. 2002;4(6):445‐450. 12021771 10.1038/ncb799

[21] Rothe M , Pan MG , Henzel WJ , Ayres TM , Goeddel DV . The TNFR2‐TRAF signaling complex contains two novel proteins related to baculoviral inhibitor of apoptosis proteins. Cell. 1995;83(7):1243‐1252. 8548810 10.1016/0092-8674(95)90149-3

[22] Du C , Fang M , Li Y , Li L , Wang X . Smac, a mitochondrial protein that promotes cytochrome c‐dependent caspase activation by eliminating IAP inhibition. Cell. 2000;102(1):33‐42. 10929711 10.1016/s0092-8674(00)00008-8

[23] Verhagen AM , Ekert PG , Pakusch M , et al. Identification of DIABLO, a mammalian protein that promotes apoptosis by binding to and antagonizing IAP proteins. Cell. 2000;102(1):43‐53. 10929712 10.1016/s0092-8674(00)00009-x

[24] Tait SW , Werner AB , de Vries E , Borst J . Mechanism of action of Drosophila reaper in mammalian cells: reaper globally inhibits protein synthesis and induces apoptosis independent of mitochondrial permeability. Cell Death Differ. 2004;11(8):800‐811. 15044965 10.1038/sj.cdd.4401410

[25] Holley CL , Olson MR , Colon‐Ramos DA , Kornbluth S . Reaper eliminates IAP proteins through stimulated IAP degradation and generalized translational inhibition. Nat Cell Biol. 2002;4(6):439‐444. 12021770 10.1038/ncb798 PMC2713440

[26] Cheng TC , Akey IV , Yuan S , Yu Z , Ludtke SJ , Akey CW . A near‐atomic structure of the dark apoptosome provides insight into assembly and activation. Structure. 2017;25(1):40‐52. 27916517 10.1016/j.str.2016.11.002 PMC5214966

[27] Means JC , Muro I , Clem RJ . Lack of involvement of mitochondrial factors in caspase activation in a Drosophila cell‐free system. Cell Death Differ. 2006;13(7):1222‐1234. 16322754 10.1038/sj.cdd.4401821 PMC2575646

[28] Dorstyn L , Mills K , Lazebnik Y , Kumar S . The two cytochrome c species, DC3 and DC4, are not required for caspase activation and apoptosis in Drosophila cells. J Cell Biol. 2004;167(3):405‐410. 15533997 10.1083/jcb.200408054 PMC2172470

[29] Igaki T , Kanuka H , Inohara N , et al. Drob‐1, a Drosophila member of the Bcl‐2/CED‐9 family that promotes cell death. Proc Natl Acad Sci USA. 2000;97(2):662‐667. 10639136 10.1073/pnas.97.2.662 PMC15387

[30] Colussi PA , Quinn LM , Huang DC , et al. Debcl, a proapoptotic Bcl‐2 homologue, is a component of the Drosophila melanogaster cell death machinery. J Cell Biol. 2000;148(4):703‐714. 10684252 10.1083/jcb.148.4.703 PMC2169366

[31] Brachmann CB , Jassim OW , Wachsmuth BD , Cagan RL . The Drosophila bcl‐2 family member dBorg‐1 functions in the apoptotic response to UV‐irradiation. Curr Biol. 2000;10(9):547‐550. 10801447 10.1016/s0960-9822(00)00474-7

[32] Sevrioukov EA , Burr J , Huang EW , et al. Drosophila Bcl‐2 proteins participate in stress‐induced apoptosis, but are not required for normal development. Genesis. 2007;45(4):184‐193. 17417787 10.1002/dvg.20279

[33] Gaumer S , Guenal I , Brun S , Theodore L , Mignotte B . Bcl‐2 and bax mammalian regulators of apoptosis are functional in drosophila. Cell Death Differ. 2000;7(9):804‐814. 11042675 10.1038/sj.cdd.4400714

[34] Abdelwahid E , Yokokura T , Krieser RJ , Balasundaram S , Fowle WH , White K . Mitochondrial disruption in Drosophila apoptosis. Dev Cell. 2007;12(5):793‐806. 17488629 10.1016/j.devcel.2007.04.004

[35] Dixon SJ , Lemberg KM , Lamprecht MR , et al. Ferroptosis: an iron‐dependent form of nonapoptotic cell death. Cell. 2012;149(5):1060‐1072. 22632970 10.1016/j.cell.2012.03.042 PMC3367386

[36] Yang WS , Kim KJ , Gaschler MM , Patel M , Shchepinov MS , Stockwell BR . Peroxidation of polyunsaturated fatty acids by lipoxygenases drives ferroptosis. Proc Natl Acad Sci USA. 2016;113(34):E4966‐E4975. 27506793 10.1073/pnas.1603244113 PMC5003261

[37] Yang WS , SriRamaratnam R , Welsch ME , et al. Regulation of ferroptotic cancer cell death by GPX4. Cell. 2014;156(1–2):317‐331. 24439385 10.1016/j.cell.2013.12.010 PMC4076414

[38] Friedmann Angeli JP , Schneider M , Proneth B , et al. Inactivation of the ferroptosis regulator Gpx4 triggers acute renal failure in mice. Nat Cell Biol. 2014;16(12):1180‐1191. 25402683 10.1038/ncb3064 PMC4894846

[39] Mishima E , Ito J , Wu Z , et al. A non‐canonical vitamin K cycle is a potent ferroptosis suppressor. Nature. 2022;608(7924):778‐783. 35922516 10.1038/s41586-022-05022-3 PMC9402432

[40] Stramer B , Wood W , Galko MJ , et al. Live imaging of wound inflammation in Drosophila embryos reveals key roles for small GTPases during in vivo cell migration. J Cell Biol. 2005;168(4):567‐573. 15699212 10.1083/jcb.200405120 PMC2171743

[41] Weavers H , Evans IR , Martin P , Wood W . Corpse engulfment generates a molecular memory that primes the macrophage inflammatory response. Cell. 2016;165(7):1658‐1671. 27212238 10.1016/j.cell.2016.04.049 PMC4912690

[42] Roddie HG , Armitage EL , Coates JA , Johnston SA , Evans IR . Simu‐dependent clearance of dying cells regulates macrophage function and inflammation resolution. PLoS Biol. 2019;17(5):e2006741. 31086359 10.1371/journal.pbio.2006741 PMC6516643

[43] Raymond MH , Davidson AJ , Shen Y , et al. Live cell tracking of macrophage efferocytosis during Drosophila embryo development in vivo. Science. 2022;375(6585):1182‐1187. 35271315 10.1126/science.abl4430 PMC7612538

[44] O'Connor JT , Stevens AC , Shannon EK , et al. Proteolytic activation of growth‐blocking peptides triggers calcium responses through the GPCR Mthl10 during epithelial wound detection. Dev Cell. 2021;56(15):2160‐2175 e2165. 34273275 10.1016/j.devcel.2021.06.020 PMC8367014

[45] O'Connor J , Akbar FB , Hutson MS , Page‐McCaw A . Zones of cellular damage around pulsed‐laser wounds. PloS One. 2021;16(9):e0253032. 34570791 10.1371/journal.pone.0253032 PMC8476025

[46] Razzell W , Evans IR , Martin P , Wood W . Calcium flashes orchestrate the wound inflammatory response through DUOX activation and hydrogen peroxide release. Curr Biol. 2013;23(5):424‐429. 23394834 10.1016/j.cub.2013.01.058 PMC3629559

[47] Niethammer P , Grabher C , Look AT , Mitchison TJ . A tissue‐scale gradient of hydrogen peroxide mediates rapid wound detection in zebrafish. Nature. 2009;459(7249):996‐999. 19494811 10.1038/nature08119 PMC2803098

[48] Moreira S , Stramer B , Evans I , Wood W , Martin P . Prioritization of competing damage and developmental signals by migrating macrophages in the Drosophila embryo. Curr Biol. 2010;20(5):464‐470. 20188558 10.1016/j.cub.2010.01.047

[49] Linkermann A , Skouta R , Himmerkus N , et al. Synchronized renal tubular cell death involves ferroptosis. Proc Natl Acad Sci USA. 2014;111(47):16836‐16841. 25385600 10.1073/pnas.1415518111 PMC4250130

[50] Katikaneni A , Jelcic M , Gerlach GF , Ma Y , Overholtzer M , Niethammer P . Lipid peroxidation regulates long‐range wound detection through 5‐lipoxygenase in zebrafish. Nat Cell Biol. 2020;22(9):1049‐1055. 32868902 10.1038/s41556-020-0564-2 PMC7898270

[51] Hu Y , Wu H , Lu C , et al. Cadmium chloride exposure impairs the growth and behavior of Drosophila via ferroptosis. Sci Total Environ. 2022;865:161183. 36581278 10.1016/j.scitotenv.2022.161183

[52] Mumbauer S , Pascual J , Kolotuev I , Hamaratoglu F . Ferritin heavy chain protects the developing wing from reactive oxygen species and ferroptosis. PLoS Genet. 2019;15(9):e1008396. 31568497 10.1371/journal.pgen.1008396 PMC6786644

[53] Johnson AG , Wein T , Mayer ML , et al. Bacterial gasdermins reveal an ancient mechanism of cell death. Science. 2022;375(6577):221‐225. 35025633 10.1126/science.abj8432 PMC9134750

[54] Harvey NL , Daish T , Mills K , et al. Characterization of the Drosophila caspase, DAMM. J Biol Chem. 2001;276(27):25342‐25350. 11337486 10.1074/jbc.M009444200

[55] Dorstyn L , Read SH , Quinn LM , Richardson H , Kumar S . DECAY, a novel Drosophila caspase related to mammalian caspase‐3 and caspase‐7. J Biol Chem. 1999;274(43):30778‐30783. 10521468 10.1074/jbc.274.43.30778

[56] Li M , Sun S , Priest J , Bi X , Fan Y . Characterization of TNF‐induced cell death in Drosophila reveals caspase‐ and JNK‐dependent necrosis and its role in tumor suppression. Cell Death Dis. 2019;10(8):613. 31409797 10.1038/s41419-019-1862-0 PMC6692325

[57] Dondelinger Y , Hulpiau P , Saeys Y , Bertrand MJM , Vandenabeele P . An evolutionary perspective on the necroptotic pathway. Trends Cell Biol. 2016;26(10):721‐732. 27368376 10.1016/j.tcb.2016.06.004

[58] Berry DL , Baehrecke EH . Growth arrest and autophagy are required for salivary gland cell degradation in Drosophila. Cell. 2007;131(6):1137‐1148. 18083103 10.1016/j.cell.2007.10.048 PMC2180345

[59] Mondragon AA , Yalonetskaya A , Ortega AJ , et al. Lysosomal machinery drives extracellular acidification to direct non‐apoptotic cell death. Cell Rep. 2019;27(1):11‐19 e13. 30943394 10.1016/j.celrep.2019.03.034 PMC6613820

[60] Etchegaray JI , Timmons AK , Klein AP , et al. Draper acts through the JNK pathway to control synchronous engulfment of dying germline cells by follicular epithelial cells. Development. 2012;139(21):4029‐4039. 22992958 10.1242/dev.082776 PMC3472587

[61] Peterson JS , McCall K . Combined inhibition of autophagy and caspases fails to prevent developmental nurse cell death in the Drosophila melanogaster ovary. PloS One. 2013;8(9):e76046. 24098761 10.1371/journal.pone.0076046 PMC3786910

[62] Lebo DPV , McCall K . Murder on the ovarian express: A tale of non‐autonomous cell death in the Drosophila ovary. Cell. 2021;10(6):1454. 10.3390/cells10061454 PMC8228772 34200604

[63] Davidson AJ , Wood W . Phagocyte responses to cell death in flies. Cold Spring Harb Perspect Biol. 2020;12(4):a03635. 10.1101/cshperspect.a036350 PMC7111249 31501193

[64] Márkus R , Laurinyecz B , Kurucz E , et al. Sessile hemocytes as a hematopoietic compartment in Drosophila melanogaster. Proc Natl Acad Sci USA. 2009;106(12):4805‐4809. 19261847 10.1073/pnas.0801766106 PMC2660760

[65] Makhijani K , Alexander B , Tanaka T , Rulifson E , Brückner K . The peripheral nervous system supports blood cell homing and survival in the Drosophila larva. Development. 2011;138(24):5379‐5391. 22071105 10.1242/dev.067322 PMC3222213

[66] Makhijani K , Alexander B , Rao D , et al. Regulation of drosophila hematopoietic sites by activin‐β from active sensory neurons. Nat Commun. 2017;8:15990. 28748922 10.1038/ncomms15990 PMC5537569

[67] Tepass U , Fessler LI , Aziz A , Hartenstein V . Embryonic origin of hemocytes and their relationship to cell death in Drosophila. Development. 1994;120(7):1829‐1837. 7924990 10.1242/dev.120.7.1829

[68] Wood W , Faria C , Jacinto A . Distinct mechanisms regulate hemocyte chemotaxis during development and wound healing in Drosophila melanogaster. J Cell Biol. 2006;173(3):405‐416. 16651377 10.1083/jcb.200508161 PMC2063841

[69] Ratheesh A , Belyaeva V , Siekhaus DE . Drosophila immune cell migration and adhesion during embryonic development and larval immune responses. Curr Opin Cell Biol. 2015;36:71‐79. 26210104 10.1016/j.ceb.2015.07.003

[70] Ratheesh A , Biebl J , Vesela J , et al. Drosophila TNF modulates tissue tension in the embryo to facilitate macrophage invasive migration. Dev Cell. 2018;45(3):331‐346 e337. 29738712 10.1016/j.devcel.2018.04.002

[71] Akhmanova M , Emtenani S , Krueger D , et al. Cell division in tissues enables macrophage infiltration. Science. 2022;376(6591):394‐396. 35446632 10.1126/science.abj0425

[72] Siekhaus D , Haesemeyer M , Moffitt O , Lehmann R . RhoL controls invasion and Rap1 localization during immune cell transmigration in Drosophila. Nat Cell Biol. 2010;12(6):605‐610. 20495554 10.1038/ncb2063 PMC3006444

[73] Davis JR , Huang CY , Zanet J , et al. Emergence of embryonic pattern through contact inhibition of locomotion. Development. 2012;139(24):4555‐4560. 23172914 10.1242/dev.082248 PMC3509721

[74] Abrams JM , White K , Fessler LI , Steller H . Programmed cell death during Drosophila embryogenesis. Development. 1993;117(1):29‐43. 8223253 10.1242/dev.117.1.29

[75] Gilbert SF . Ecological developmental biology: environmental signals for normal animal development. Evol Dev. 2012;14(1):20‐28. 23016971 10.1111/j.1525-142X.2011.00519.x

[76] Bangs P , Franc N , White K . Molecular mechanisms of cell death and phagocytosis in drosophila. Cell Death Differ. 2000;7(11):1027‐1034. 11139274 10.1038/sj.cdd.4400754

[77] Rogulja‐Ortmann A , Lüer K , Seibert J , Rickert C , Technau GM . Programmed cell death in the embryonic central nervous system of Drosophila melanogaster. Development. 2007;134(1):105‐116. 17164416 10.1242/dev.02707

[78] Kurant E , Axelrod S , Leaman D , Gaul U . Six‐microns‐under acts upstream of Draper in the glial phagocytosis of apoptotic neurons. Cell. 2008;133(3):498‐509. 18455990 10.1016/j.cell.2008.02.052 PMC2730188

[79] Hanisch UK , Kettenmann H . Microglia: active sensor and versatile effector cells in the normal and pathologic brain. Nat Neurosci. 2007;10(11):1387‐1394. 17965659 10.1038/nn1997

[80] Cunningham CL , Martínez‐Cerdeño V , Noctor SC . Microglia regulate the number of neural precursor cells in the developing cerebral cortex. J Neurosci. 2013;33(10):4216‐4233. 23467340 10.1523/JNEUROSCI.3441-12.2013 PMC3711552

[81] Cronk JC , Kipnis J . Microglia—the brain's busy bees. F1000Prime Rep. 2013;5:53. 24381729 10.12703/P5-53 PMC3854698

[82] Bilimoria PM , Stevens B . Microglia function during brain development: new insights from animal models. Brain Res. 2015;1617:7‐17. 25463024 10.1016/j.brainres.2014.11.032

[83] Casano AM , Peri F . Microglia: multitasking specialists of the brain. Dev Cell. 2015;32(4):469‐477. 25710533 10.1016/j.devcel.2015.01.018

[84] Ziegenfuss JS , Biswas R , Avery MA , et al. Draper‐dependent glial phagocytic activity is mediated by Src and Syk family kinase signalling. Nature. 2008;453(7197):935‐939. 18432193 10.1038/nature06901 PMC2493287

[85] Tasdemir‐Yilmaz OE , Freeman MR . Astrocytes engage unique molecular programs to engulf pruned neuronal debris from distinct subsets of neurons. Genes Dev. 2014;28(1):20‐33. 24361692 10.1101/gad.229518.113 PMC3894410

[86] Nakano R , Iwamura M , Obikawa A , et al. Cortex glia clear dead young neurons via Drpr/dCed‐6/Shark and Crk/Mbc/dCed‐12 signaling pathways in the developing Drosophila optic lobe. Dev Biol. 2019;453(1):68‐85. 31063730 10.1016/j.ydbio.2019.05.003

[87] Freeman MR , Doherty J . Glial cell biology in Drosophila and vertebrates. Trends Neurosci. 2006;29(2):82‐90. 16377000 10.1016/j.tins.2005.12.002

[88] Logan MA , Freeman MR . The scoop on the fly brain: glial engulfment functions in Drosophila. Neuron Glia Biol. 2007;3(1):63‐74. 18172512 10.1017/S1740925X07000646 PMC2171361

[89] Kurant E . Keeping the CNS clear: glial phagocytic functions in Drosophila. Glia. 2011;59(9):1304‐1311. 21136555 10.1002/glia.21098

[90] Logan MA . Glial contributions to neuronal health and disease: new insights from Drosophila. Curr Opin Neurobiol. 2017;47:162‐167. 29096245 10.1016/j.conb.2017.10.008 PMC5741183

[91] Sonnenfeld MJ , Jacobs JR . Macrophages and glia participate in the removal of apoptotic neurons from the Drosophila embryonic nervous system. J Comp Neurol. 1995;359(4):644‐652. 7499553 10.1002/cne.903590410

[92] Cantera R , Technau GM . Glial cells phagocytose neuronal debris during the metamorphosis of the central nervous system in Drosophila melanogaster. Dev Genes Evol. 1996;206(4):277‐280. 24173566 10.1007/s004270050052

[93] Freeman MR , Delrow J , Kim J , Johnson E , Doe CQ . Unwrapping glial biology: Gcm target genes regulating glial development, diversification, and function. Neuron. 2003;38(4):567‐580. 12765609 10.1016/s0896-6273(03)00289-7

[94] Shklyar B , Sellman Y , Shklover J , Mishnaevski K , Levy‐Adam F , Kurant E . Developmental regulation of glial cell phagocytic function during Drosophila embryogenesis. Dev Biol. 2014;393(2):255‐269. 25046770 10.1016/j.ydbio.2014.07.005

[95] Hilu‐Dadia R , Kurant E . Glial phagocytosis in developing and mature Drosophila CNS: tight regulation for a healthy brain. Curr Opin Immunol. 2020;62:62‐68. 31862622 10.1016/j.coi.2019.11.010

[96] Armitage EL , Roddie HG , Evans IR . Overexposure to apoptosis via disrupted glial specification perturbs Drosophila macrophage function and reveals roles of the CNS during injury. Cell Death Dis. 2020;11(8):627. 32796812 10.1038/s41419-020-02875-2 PMC7428013

[97] Trebuchet G , Cattenoz PB , Zsamboki J , et al. The repo homeodomain transcription factor suppresses hematopoiesis in Drosophila and preserves the glial fate. J Neurosci. 2019;39(2):238‐255. 30504274 10.1523/JNEUROSCI.1059-18.2018 PMC6360283

[98] Evans IR , Rodrigues FS , Armitage EL , Wood W . Draper/CED‐1 mediates an ancient damage response to control inflammatory blood cell migration in vivo. Curr Biol. 2015;25(12):1606‐1612. 26028435 10.1016/j.cub.2015.04.037 PMC4503800

[99] Ayoub M , David LM , Shklyar B , Hakim‐Mishnaevski K , Kurant E . Drosophila FGFR/Htl signaling shapes embryonic glia to phagocytose apoptotic neurons. Cell Death Discov. 2023;9(1):90. 36898998 10.1038/s41420-023-01382-5 PMC10006210

[100] Franc NC , Heitzler P , Ezekowitz RA , White K . Requirement for croquemort in phagocytosis of apoptotic cells in Drosophila. Science. 1999;284(5422):1991‐1994. 10373118 10.1126/science.284.5422.1991

[101] Shklover J , Mishnaevski K , Levy‐Adam F , Kurant E . JNK pathway activation is able to synchronize neuronal death and glial phagocytosis in Drosophila. Cell Death Dis. 2015;6(2):e1649. 25695602 10.1038/cddis.2015.27 PMC4669801

[102] Awasaki T , Tatsumi R , Takahashi K , et al. Essential role of the apoptotic cell engulfment genes draper and ced‐6 in programmed axon pruning during Drosophila metamorphosis. Neuron. 2006;50(6):855‐867. 16772168 10.1016/j.neuron.2006.04.027

[103] Williams DW , Kondo S , Krzyzanowska A , Hiromi Y , Truman JW . Local caspase activity directs engulfment of dendrites during pruning. Nat Neurosci. 2006;9(10):1234‐1236. 16980964 10.1038/nn1774

[104] Corty MM , Freeman MR . Cell biology in neuroscience: architects in neural circuit design: glia control neuron numbers and connectivity. J Cell Biol. 2013;203(3):395‐405. 24217617 10.1083/jcb.201306099 PMC3824021

[105] Davidson AJ , Wood W . Macrophages use distinct Actin regulators to switch engulfment strategies and ensure phagocytic plasticity In vivo. Cell Rep. 2020;31(8):107692. 32460022 10.1016/j.celrep.2020.107692 PMC7262594

[106] Ball JA , Vlisidou I , Blunt MD , Wood W , Ward SG . Hydrogen peroxide triggers a dual signaling Axis to selectively suppress activated human T lymphocyte migration. J Immunol. 2017;198(9):3679‐3689. 28363904 10.4049/jimmunol.1600868 PMC5392728

[107] Pravda J . Systemic lupus erythematosus: pathogenesis at the functional limit of redox homeostasis. Oxid Med Cell Longev. 2019;2019:1651724. 31885772 10.1155/2019/1651724 PMC6899283

[108] Williamson AP , Vale RD . Spatial control of Draper receptor signaling initiates apoptotic cell engulfment. J Cell Biol. 2018;217(11):3977‐3992. 30139739 10.1083/jcb.201711175 PMC6219719

[109] Campbell JS , Davidson AJ , Todd H , et al. PTPN21/Pez is a novel and evolutionarily conserved key regulator of inflammation In vivo. Curr Biol. 2020;31:875‐883.e5. 33296680 10.1016/j.cub.2020.11.014 PMC7902905

[110] Logan MA , Hackett R , Doherty J , Sheehan A , Speese SD , Freeman MR . Negative regulation of glial engulfment activity by Draper terminates glial responses to axon injury. Nat Neurosci. 2012;15(5):722‐730. 22426252 10.1038/nn.3066 PMC3337949

[111] Cattenoz PB , Sakr R , Pavlidaki A , et al. Temporal specificity and heterogeneity of drosophila immune cells. EMBO J. 2020;39(12):e104486. 32162708 10.15252/embj.2020104486 PMC7298292

[112] Tattikota SG , Cho B , Liu Y , et al. A single‐cell survey of Drosophila blood. eLife. 2020;9:e54818. 32396065 10.7554/eLife.54818 PMC7237219

[113] Cho B , Yoon SH , Lee D , et al. Single‐cell transcriptome maps of myeloid blood cell lineages in Drosophila. Nat Commun. 2020;11(1):4483. 32900993 10.1038/s41467-020-18135-y PMC7479620

[114] Fu Y , Huang X , Zhang P , van de Leemput J , Han Z . Single‐cell RNA sequencing identifies novel cell types in Drosophila blood. J Genet Genomics. 2020;47(4):175‐186. 32487456 10.1016/j.jgg.2020.02.004 PMC7321924

[115] Coates JA , Brooks E , Brittle AL , Armitage EL , Zeidler MP , Evans IR . Identification of functionally distinct macrophage subpopulations in Drosophila. eLife. 2021;10:e58686. 33885361 10.7554/eLife.58686 PMC8062135

[116] Omoto JJ , Yogi P , Hartenstein V . Origin and development of neuropil glia of the Drosophila larval and adult brain: two distinct glial populations derived from separate progenitors. Dev Biol. 2015;404(2):2‐20. 25779704 10.1016/j.ydbio.2015.03.004 PMC4515183

[117] Omoto JJ , Lovick JK , Hartenstein V . Origins of glial cell populations in the insect nervous system. Curr Opin Insect Sci. 2016;18:96‐104. 27939718 10.1016/j.cois.2016.09.003 PMC5825180

[118] Doherty J , Logan MA , Taşdemir OE , Freeman MR . Ensheathing glia function as phagocytes in the adult Drosophila brain. J Neurosci. 2009;29(15):4768‐4781. 19369546 10.1523/JNEUROSCI.5951-08.2009 PMC2674269

[119] Timmons AK , Mondragon AA , Schenkel CE , et al. Phagocytosis genes nonautonomously promote developmental cell death in the Drosophila ovary. Proc Natl Acad Sci USA. 2016;113(9):E1246‐E1255. 26884181 10.1073/pnas.1522830113 PMC4780630

[120] Santoso CS , Meehan TL , Peterson JS , Cedano TM , Turlo CV , McCall K . The ABC transporter. G3 (Bethesda). 2018;8(3):833‐843. 29295819 10.1534/g3.117.300427 PMC5844305

[121] Franz A , Wood W , Martin P . Fat body cells are motile and actively migrate to wounds to drive repair and prevent infection. Dev Cell. 2018;44(4):460‐470.e463. 29486196 10.1016/j.devcel.2018.01.026 PMC6113741

[122] Nimmerjahn A , Kirchhoff F , Helmchen F . Resting microglial cells are highly dynamic surveillants of brain parenchyma in vivo. Science. 2005;308(5726):1314‐1318. 15831717 10.1126/science.1110647

[123] Moller K , Brambach M , Villani A , Gallo E , Gilmour D , Peri F . A role for the centrosome in regulating the rate of neuronal efferocytosis by microglia in vivo. eLife. 2022;11:e82094 36398880 10.7554/eLife.82094 PMC9674339

[124] Svitkina TM , Borisy GG . Arp2/3 Complex and Actin depolymerizing factor/cofilin in dendritic organization and treadmilling of Actin filament array in lamellipodia. J Cell Biol. 1999;145(5):1009‐1026. 10352018 10.1083/jcb.145.5.1009 PMC2133125

[125] Mullins RD , Heuser JA , Pollard TD . The interaction of Arp2/3 complex with Actin: nucleation, high affinity pointed end capping, and formation of branching networks of filaments. Proc Natl Acad Sci USA. 1998;95(11):6181‐6186. 9600938 10.1073/pnas.95.11.6181 PMC27619

[126] Machesky LM , Mullins RD , Higgs HN , et al. Scar, a WASp‐related protein, activates nucleation of actin filaments by the Arp2/3 complex. Proc Natl Acad Sci USA. 1999;96(7):3739‐3744. 10097107 10.1073/pnas.96.7.3739 PMC22364

[127] Yolland L , Burki M , Marcotti S , et al. Persistent and polarized global Actin flow is essential for directionality during cell migration. Nat Cell Biol. 2019;21(11):1370‐1381. 31685997 10.1038/s41556-019-0411-5 PMC7025891

[128] Zanet J , Stramer B , Millard T , Martin P , Payre F , Plaza S . Fascin is required for blood cell migration during Drosophila embryogenesis. Development. 2009;136(15):2557‐2565. 19592575 10.1242/dev.036517

[129] Tucker PK , Evans IR , Wood W . Ena drives invasive macrophage migration in Drosophila embryos. Dis Model Mech. 2011;4(1):126‐134. 21045209 10.1242/dmm.005694 PMC3008967

[130] Davidson AJ , Millard TH , Evans IR , Wood W . Ena orchestrates remodelling within the actin cytoskeleton to drive robust Drosophila macrophage chemotaxis. J Cell Sci. 2019;132(5):jcs224618. 30718364 10.1242/jcs.224618 PMC6432709

[131] Winkelman JD , Bilancia CG , Peifer M , Kovar DR . Ena/VASP enabled is a highly processive actin polymerase tailored to self‐assemble parallel‐bundled F‐actin networks with Fascin. Proc Natl Acad Sci USA. 2014;111(11):4121‐4126. 24591594 10.1073/pnas.1322093111 PMC3964058

[132] Bilancia CG , Winkelman JD , Tsygankov D , et al. Enabled negatively regulates diaphanous‐driven actin dynamics in vitro and in vivo. Dev Cell. 2014;28(4):394‐408. 24576424 10.1016/j.devcel.2014.01.015 PMC3992947

[133] Homem CC , Peifer M . Exploring the roles of diaphanous and enabled activity in shaping the balance between filopodia and lamellipodia. Mol Biol Cell. 2009;20(24):5138‐5155. 19846663 10.1091/mbc.E09-02-0144 PMC2793291

[134] Rotty JD , Brighton HE , Craig SL , et al. Arp2/3 complex is required for macrophage integrin functions but is dispensable for FcR phagocytosis and In vivo motility. Dev Cell. 2017;42(5):498‐513 e496. 28867487 10.1016/j.devcel.2017.08.003 PMC5601320

[135] Evans IR , Ghai PA , Urbancic V , Tan KL , Wood W . SCAR/WAVE‐mediated processing of engulfed apoptotic corpses is essential for effective macrophage migration in Drosophila. Cell Death Differ. 2013;20(5):709‐720. 23328632 10.1038/cdd.2012.166 PMC3619236

[136] Wu C , Asokan SB , Berginski ME , et al. Arp2/3 is critical for lamellipodia and response to extracellular matrix cues but is dispensable for chemotaxis. Cell. 2012;148(5):973‐987. 22385962 10.1016/j.cell.2011.12.034 PMC3707508

[137] Davidson AJ , Amato C , Thomason PA , Insall RH . WASP family proteins and formins compete in pseudopod‐ and bleb‐based migration. J Cell Biol. 2018;217(2):701‐714. 29191847 10.1083/jcb.201705160 PMC5800805

[138] Suraneni P , Rubinstein B , Unruh JR , Durnin M , Hanein D , Li R . The Arp2/3 complex is required for lamellipodia extension and directional fibroblast cell migration. J Cell Biol. 2012;197(2):239‐251. 22492726 10.1083/jcb.201112113 PMC3328382

[139] Evans IR , Hu N , Skaer H , Wood W . Interdependence of macrophage migration and ventral nerve cord development in Drosophila embryos. Development. 2010;137(10):1625‐1633. 20392742 10.1242/dev.046797 PMC2860247

[140] Paterson N , Lammermann T . Macrophage network dynamics depend on haptokinesis for optimal local surveillance. eLife. 2022;11:e75354. 35343899 10.7554/eLife.75354 PMC8963880

[141] Uderhardt S , Martins AJ , Tsang JS , Lammermann T , Germain RN . Resident macrophages cloak tissue microlesions to prevent neutrophil‐driven inflammatory damage. Cell. 2019;177(3):541‐555 e517. 30955887 10.1016/j.cell.2019.02.028 PMC6474841

[142] Barth ND , Marwick JA , Vendrell M , Rossi AG , Dransfield I . The “phagocytic synapse” and clearance of apoptotic cells. Front Immunol. 2017;8:1708. 29255465 10.3389/fimmu.2017.01708 PMC5723006

[143] Fond AM , Ravichandran KS . Clearance of dying cells by phagocytes: mechanisms and implications for disease pathogenesis. Adv Exp Med Biol. 2016;930:25‐49. 27558816 10.1007/978-3-319-39406-0_2 PMC6721615

[144] Meehan TL , Kleinsorge SE , Timmons AK , Taylor JD , McCall K . Polarization of the epithelial layer and apical localization of integrins are required for engulfment of apoptotic cells in the Drosophila ovary. Dis Model Mech. 2015;8(12):1603‐1614. 26398951 10.1242/dmm.021998 PMC4728319

[145] Kurucz E , Markus R , Zsamboki J , et al. Nimrod, a putative phagocytosis receptor with EGF repeats in Drosophila plasmatocytes. Curr Biol. 2007;17(7):649‐654. 17363253 10.1016/j.cub.2007.02.041

[146] Somogyi K , Sipos B , Penzes Z , et al. Evolution of genes and repeats in the Nimrod superfamily. Mol Biol Evol. 2008;25(11):2337‐2347. 18703524 10.1093/molbev/msn180

[147] Melcarne C , Ramond E , Dudzic J , et al. Two nimrod receptors, NimC1 and eater, synergistically contribute to bacterial phagocytosis in Drosophila melanogaster. FEBS J. 2019;286(14):2670‐2691. 30993828 10.1111/febs.14857 PMC6852320

[148] MacDonald JM , Beach MG , Porpiglia E , Sheehan AE , Watts RJ , Freeman MR . The Drosophila cell corpse engulfment receptor Draper mediates glial clearance of severed axons. Neuron. 2006;50(6):869‐881. 16772169 10.1016/j.neuron.2006.04.028

[149] Shklyar B , Levy‐Adam F , Mishnaevski K , Kurant E . Caspase activity is required for engulfment of apoptotic cells. Mol Cell Biol. 2013;33(16):3191‐3201. 23754750 10.1128/MCB.00233-13 PMC3753910

[150] Tung TT , Nagaosa K , Fujita Y , et al. Phosphatidylserine recognition and induction of apoptotic cell clearance by Drosophila engulfment receptor Draper. J Biochem. 2013;153(5):483‐491. 23420848 10.1093/jb/mvt014

[151] Petrignani B , Rommelaere S , Hakim‐Mishnaevski K , et al. A secreted factor NimrodB4 promotes the elimination of apoptotic corpses by phagocytes in Drosophila. EMBO Rep. 2021;22(9):e52262. 34370384 10.15252/embr.202052262 PMC8419693

[152] Manaka J , Kuraishi T , Shiratsuchi A , et al. Draper‐mediated and phosphatidylserine‐independent phagocytosis of apoptotic cells by Drosophila hemocytes/macrophages. J Biol Chem. 2004;279(46):48466‐48476. 15342648 10.1074/jbc.M408597200

[153] Ji H , Wang B , Shen Y , et al. The Drosophila chemokine‐like Orion bridges phosphatidylserine and Draper in phagocytosis of neurons. Proc Natl Acad Sci USA. 2023;120(24):e2303392120. 37276397 10.1073/pnas.2303392120 PMC10268242

[154] Kinchen JM , Doukoumetzidis K , Almendinger J , et al. A pathway for phagosome maturation during engulfment of apoptotic cells. Nat Cell Biol. 2008;10(5):556‐566. 18425118 10.1038/ncb1718 PMC2851549

[155] Garg A , Wu LP . Drosophila Rab14 mediates phagocytosis in the immune response to *Staphylococcus aureus* . Cell Microbiol. 2014;16(2):296‐310. 24119134 10.1111/cmi.12220 PMC4120862

[156] Cheng LW , Viala JP , Stuurman N , Wiedemann U , Vale RD , Portnoy DA . Use of RNA interference in Drosophila S2 cells to identify host pathways controlling compartmentalization of an intracellular pathogen. Proc Natl Acad Sci USA. 2005;102(38):13646‐13651. 16157870 10.1073/pnas.0506461102 PMC1224656

[157] Philips JA , Rubin EJ , Perrimon N . Drosophila RNAi screen reveals CD36 family member required for mycobacterial infection. Science. 2005;309(5738):1251‐1253. 16020694 10.1126/science.1116006

[158] Carnell M , Zech T , Calaminus SD , et al. Actin polymerization driven by WASH causes V‐ATPase retrieval and vesicle neutralization before exocytosis. J Cell Biol. 2011;193(5):831‐839. 21606208 10.1083/jcb.201009119 PMC3105540

[159] Kolonko M , Geffken AC , Blumer T , Hagens K , Schaible UE , Hagedorn M . WASH‐driven actin polymerization is required for efficient mycobacterial phagosome maturation arrest. Cell Microbiol. 2014;16(2):232‐246. 24119059 10.1111/cmi.12217

[160] Nagel BM , Bechtold M , Rodriguez LG , Bogdan S . Drosophila WASH is required for integrin‐mediated cell adhesion, cell motility and lysosomal neutralization. J Cell Sci. 2017;130(2):344‐359. 27884932 10.1242/jcs.193086

[161] Gomez TS , Gorman JA , de Narvajas AA , Koenig AO , Billadeau DD . Trafficking defects in WASH‐knockout fibroblasts originate from collapsed endosomal and lysosomal networks. Mol Biol Cell. 2012;23(16):3215‐3228. 22718907 10.1091/mbc.E12-02-0101 PMC3418315

[162] Sanjuan MA , Dillon CP , Tait SW , et al. Toll‐like receptor signalling in macrophages links the autophagy pathway to phagocytosis. Nature. 2007;450(7173):1253‐1257. 18097414 10.1038/nature06421

[163] Martinez J , Almendinger J , Oberst A , et al. Microtubule‐associated protein 1 light chain 3 alpha (LC3)‐associated phagocytosis is required for the efficient clearance of dead cells. Proc Natl Acad Sci USA. 2011;108(42):17396‐17401. 21969579 10.1073/pnas.1113421108 PMC3198353

[164] Fletcher K , Ulferts R , Jacquin E , et al. The WD40 domain of ATG16L1 is required for its non‐canonical role in lipidation of LC3 at single membranes. EMBO J. 2018;37(4):e97840. 29317426 10.15252/embj.201797840 PMC5813257

[165] Martinez J , Malireddi RK , Lu Q , et al. Molecular characterization of LC3‐associated phagocytosis reveals distinct roles for Rubicon, NOX2 and autophagy proteins. Nat Cell Biol. 2015;17(7):893‐906. 26098576 10.1038/ncb3192 PMC4612372

[166] Cunha LD , Yang M , Carter R , et al. LC3‐associated phagocytosis in myeloid cells promotes tumor immune tolerance. Cell. 2018;175(2):429‐441 e416. 30245008 10.1016/j.cell.2018.08.061 PMC6201245

[167] Villani A , Benjaminsen J , Moritz C , et al. Clearance by microglia depends on packaging of phagosomes into a unique cellular compartment. Dev Cell. 2019;49(1):77‐88 e77. 30880002 10.1016/j.devcel.2019.02.014

[168] Szabo A , Vincze V , Chhatre AS , et al. LC3‐associated phagocytosis promotes glial degradation of axon debris after injury in Drosophila models. Nat Commun. 2023;14(1):3077. 37248218 10.1038/s41467-023-38755-4 PMC10227080

[169] Clemente GD , Weavers H . A PI3K‐calcium‐Nox axis primes leukocyte Nrf2 to boost immune resilience and limit collateral damage. J Cell Biol. 2023;222(6):e202203062. 36995284 10.1083/jcb.202203062 PMC10067972

[170] Meagher LC , Savill JS , Baker A , Fuller RW , Haslett C . Phagocytosis of apoptotic neutrophils does not induce macrophage release of thromboxane B2. J Leukoc Biol. 1992;52(3):269‐273. 1522386

